# Genetic associations of neuropathic pain and sensory profile in a deeply phenotyped neuropathy cohort

**DOI:** 10.1097/j.pain.0000000000003463

**Published:** 2024-10-29

**Authors:** Mikael Åkerlund, Georgios Baskozos, Wenqianglong Li, Andreas C. Themistocleous, Mathilde M.V. Pascal, N. William Rayner, Nadine Attal, Ralf Baron, Sophie Baudic, Kristine Bennedsgaard, Didier Bouhassira, Maddalena Comini, Geert Crombez, Catharina G. Faber, Nanna B. Finnerup, Janne Gierthmühlen, Yelena Granovsky, Sandra Sif Gylfadottir, Harry L. Hébert, Troels S. Jensen, Jishi John, Harriet I. Kemp, Giuseppe Lauria, Helen Laycock, Weihua Meng, Kristian Bernhard Nilsen, Colin Palmer, Andrew S.C. Rice, Jordi Serra, Blair H. Smith, Solomon Tesfaye, Leah Shafran Topaz, Abirami Veluchamy, Jan Vollert, David Yarnitsky, Natalie van Zuydam, John Anker Zwart, Mark I. McCarthy, Valeriya Lyssenko, David L. Bennett

**Affiliations:** a Department of Clinical Sciences, Lund University Diabetes Centre, Lund University, Lund, Sweden; b Nuffield Department of Clinical Neuroscience, The University of Oxford, Oxford, United Kingdom; c NIHR Oxford Biomedical Research Centre, Oxford University Hospitals Trust, Oxford, United Kingdom; d Wellcome Centre for Human Genetics, Nuffield Department of Medicine, University of Oxford, Oxford, United Kingdom; e Oxford Centre for Diabetes, Endocrinology and Metabolism, Radcliffe Department of Medicine, University of Oxford, Oxford, United Kingdom; f Department of Human Genetics, Wellcome Sanger Institute, Hinxton, United Kingdom; g Institute of Translational Genomics, Helmholtz Zentrum München, German Research Center for Environmental Health, Neuherberg, Germany; h INSERM U987, APHP and UVSQ Paris Saclay University, CHU Ambroise Paré, Boulogne Billancourt, France; i Division of Neurological Pain Research and Therapy, Department of Neurology, Universitätsklinikum Schleswig-Holstein, Kiel, Germany; j Department of Oncology, Aarhus University Hospital, Aarhus, Denmark; k Department of Experimental-Clinical and Health Psychology, Ghent University, Ghent, Belgium; l Department of Neurology, Maastricht University Medical Center, Mental Health and Neuroscience Reseach Institute, Maastricht, the Netherlands; m Department of Clinical Medicine, Danish Pain Research Center, Aarhus University, Aarhus, Denmark; n Department of Neurology, Aarhus University Hospital, Aarhus, Denmark; o Department for Anesthesiology and Surgical Intensive Care Medicine, Pain Therapy, University Hospital of Kiel, Kiel, Germany; p Department of Neurology, Rambam Health Care Campus, Technion-Israel Institute of Technology, Haifa, Israel; q Chronic Pain Research Group, Division of Population Health and Genomics, Ninewells Hospital and Medical School, University of Dundee, Dundee, United Kingdom; r Pain Research, Department of Surgery and Cancer, Faculty of Medicine, Imperial College London, London, United Kingdom; s Department of Medical Biotechnology and Translational Medicine, University of Milan, Milan, Italy; t Department of Clinical Neurosciences, IRCCS Fondazione Istituto Neurologico “Carlo Besta,” Milan, Italy; u Nottingham Ningbo China Beacons of Excellence Research and Innovation Institute, University of Nottingham Ningbo China, Ningbo, China; v Section for Clinical Neurophysiology, Department of Neurology, Oslo University Hospital, Oslo, Norway; w Division of Population Health and Genomics, Ninewells Hospital and Medical School, University of Dundee, Dundee, United Kingdom; x Department of Clinical Neurophysiology, King's College Hospital, London, United Kingdom; y Diabetes Research Unit, Sheffield Teaching Hospitals NHS Foundation Trust, Sheffield, United Kingdom; z Rappaport Faculty of Medicine, Technion-Israel Institute of Technology, Haifa, Israel; aa Department of Clinical and Biomedical Sciences, Faculty of Health and Life Sciences, University of Exeter, Exeter, United Kingdom; bb Department of Research and Innovation, Oslo University Hospital and University of Oslo, Oslo, Norway; cc Department of Clinical Science, University of Bergen, Bergen, Norway

**Keywords:** Neuropathic pain, Neuropathy, GWAS, Diabetes mellitus, *SCN9A*, Sensory profile

## Abstract

We aimed to investigate the genetic associations of neuropathic pain in a deeply phenotyped cohort. Participants with neuropathic pain were cases and compared with those exposed to injury or disease but without neuropathic pain as control subjects. Diabetic polyneuropathy was the most common aetiology of neuropathic pain. A standardised quantitative sensory testing protocol was used to categorize participants based on sensory profile. We performed genome-wide association study, and in a subset of participants, we undertook whole-exome sequencing targeting analyses of 45 known pain-related genes. In the genome-wide association study of diabetic neuropathy (N = 1541), a top significant association was found at the *KCNT2* locus linked with pain intensity (rs114159097, *P* = 3.55 × 10^−8^). Gene-based analysis revealed significant associations between *LHX8* and *TCF7L2* and neuropathic pain. Polygenic risk score for depression was associated with neuropathic pain in all participants. Polygenic risk score for C-reactive protein showed a positive association, while that for fasting insulin showed a negative association with neuropathic pain, in individuals with diabetic polyneuropathy. Gene burden analysis of candidate pain genes supported significant associations between rare variants in *SCN9A* and *OPRM1* and neuropathic pain. Comparison of individuals with the “irritable” nociceptor profile to those with a “nonirritable” nociceptor profile identified a significantly associated variant (rs72669682, *P* = 4.39 × 10^−8^) within the *ANK2* gene. Our study on a deeply phenotyped cohort with neuropathic pain has confirmed genetic associations with the known pain-related genes *KCNT2*, *OPRM1*, and *SCN9A* and identified novel associations with *LHX8* and *ANK2*, genes not previously linked to pain and sensory profiles, respectively.

## Abstract 2

Supplemental Digital Content is Available in the Text.

Studying a deeply phenotyped neuropathic pain cohort, confirmed genetic associations with known pain-related genes and identified novel associations with genes linked to pain and sensory profile.

## 1. Introduction

Neuropathic pain arises as a consequence of a disease or lesion of the somatosensory nervous system,^29^ affects 7% to 10% of the general population and has a major negative impact on quality of life.^3,6,55^ Better understanding of genetic determinants of neuropathic pain could aid patient stratification, risk prediction, treatment targeting and ultimately the development of novel treatment approaches.^11^

There are rare Mendelian pain disorders with extreme pain phenotypes such as inherited erythromelalgia (IEM) and paroxysmal extreme pain disorder (PEPD) because of monoallelic variants in *SCN9a* causing gain of function of the voltage-gated sodium channel Na_V_1.7.^8,14,21,35,73^ At a population level, neuropathic pain more commonly arises after disease or injury to the nervous system such as diabetic neuropathy, Herpes Zoster or trauma/surgery rather than a primary genetic cause. The risk and severity of this type of neuropathic pain involves a complex interplay between the severity of the disease/injury, environmental context, and multiple genes.^67^ A recent twins study provides evidence for a substantial heritable component (37%) to pain with neuropathic features.^45^

A recent systematic review^64^ (recently updated)^61^ of genetic risk factors for neuropathic pain found that most studies were candidate gene association studies of genes involved in immune responses, neurotransmission, ion channels, protein binding, receptor signalling, and metabolism. These included genes such as *SCN9A* implicated in idiopathic small fibre neuropathy^19^ and painful diabetic neuropathy^10^ as well as Mendelian pain disorders. There have been relatively few genome-wide association studies (GWASs) conducted in neuropathic pain. Those performed in diabetic populations have found suggestive variants for neuropathic pain including one near *GFRA2* (encoding GDNF family receptor alpha 2).^42,43^ A meta-analysis of GWASs of sciatica identified a genome-wide significant locus near *NFIB* (nuclear factor I B).^33^ A recent meta-analysis of GWASs of neuropathic pain revealed a genome-wide significant locus at chromosome 12q23.1, which mapped to *SLC25A3* encoding a mitochondrial phosphate carrier.^65^

The genetic epidemiology of neuropathic pain disorders presents a number of challenges, and recent efforts have been undertaken to enhance precision of the case definition, classification and grading of neuropathic pain, and of relevant controls for genetic studies. The integration of questionnaire-based neuropathic pain case definitions with clinical examination and tests of sensory function such as quantitative sensory testing (QST) strives to enhance certainty of identifying a lesion of the somatosensory nervous system.^23,63^

Quantitative sensory testing assessment across a range of modalities (using a standardised protocol)^50^ can be used to diagnose a lesion of the sensory nervous system and also generate an individual sensory profile. Such sensory profiles can then be used to stratify patients into groups that likely reflect common underlying disease mechanisms and may be predictive of treatment response.^4^ Candidate gene analysis has revealed associations with sensory profiles^9,51^ but a GWAS for specific sensory profiles has not previously been undertaken.

DOLORisk is a European consortium dedicated to investigating risk factors and determinants of neuropathic pain.^47^ We collaborated and harmonised phenotyping with other neuropathic pain consortia (GeNeup, OPTION, and PROPANE) with the aim of determining the genetic associations of neuropathic pain (including rare and common variants) in a well-defined cohort.

## 2. Methods

### 2.1. Participant recruitment and criteria for inclusion in genome-wide association study and whole exome sequencing studies

**Figure 1.**
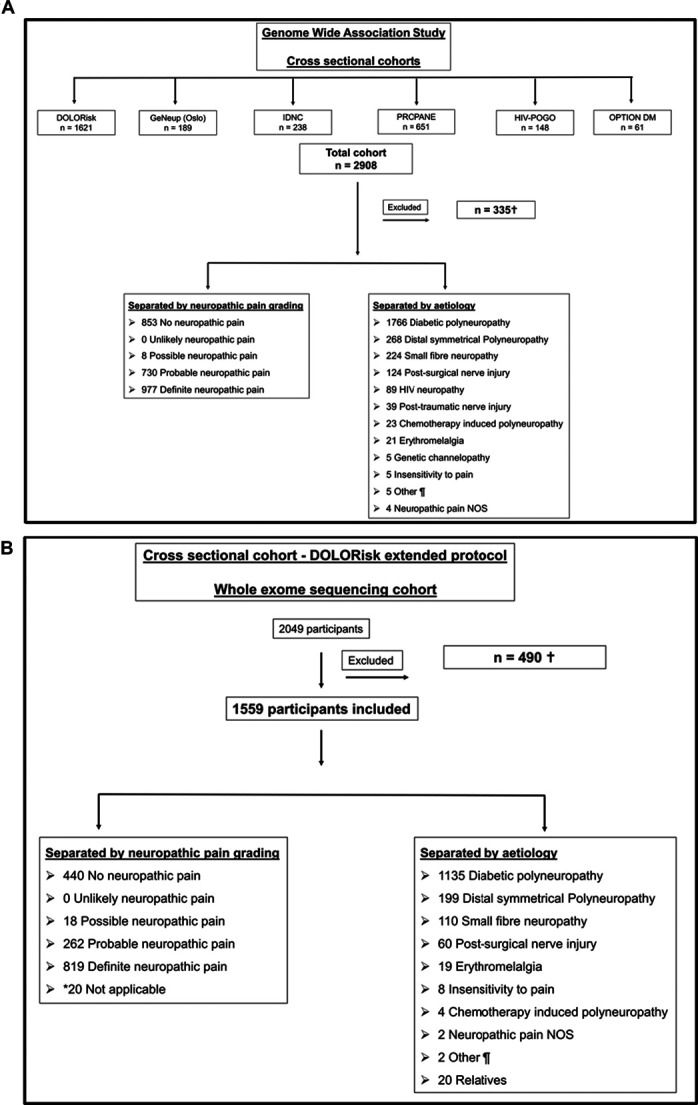
Cohorts, recruitment pathways, inclusion, exclusion. (A) Flow diagram for participant recruitment for those included in genome-wide association study. IDNC and GeNeup were included as part of DOLORisk extended cohort as clinical phenotyping was highly aligned from inception with the DOLORisk cohort. Exclusions included those with no blood samples available at the time of genotyping; no or possible neuropathy; incomplete phenotype information; unaffected relatives; failed quality control; and duplicates. Neuropathic Pain NOS is defined as pain with a distinct neuroanatomically plausible distribution; however, no evidence of nerve injury found on clinical examination or specialised investigations. ¶Other diagnosis: neuropathic itch, lumbar radiculopathy, sciatica. (B) Flow diagram for participant recruitment for those included in whole exome sequencing. Participants with a potential Mendelian basis for rare and extreme cases of neuropathic pain were recruited, and unaffected family members were invited to participate (in case they were needed for future studies to understand segregation of variants in pedigrees) but were not included in this study's analysis and unaffected family members were excluded. Exclusions included: n = 338—no blood samples available; n = 76—failed genotyping quality control; n = 71—non-Europeans; n = 4—incomplete phenotype information. *Clinical phenotyping was not performed for the 20 unaffected relatives. ¶Other diagnosis: neuropathic itch and lumbar radiculopathy.

Participants included in the GWAS and whole exome sequencing (WES) studies were recruited from the DOLORisk study^47^ and supplemented with additional cohorts (Supplementary Table 1, available at http://links.lww.com/PAIN/C169 and Fig. 1). The DOLORisk study was a multicentre observational study to understand the risk factors and determinants of neuropathic pain. A standardised protocol was used across all participating centres to identify and characterise patients with or at risk of neuropathic pain, for instance those with peripheral neuropathy.^47^ The protocol was based on recent international consensus on phenotyping neuropathic pain (NeuroPPIC),^63^ led by the Special Interest Group on Neuropathic Pain of the International Association for the Study of Pain (NeuPSIG). The instruments were chosen at a consensus meeting of all the recruitment centres. These included: standardised questionnaires to assess pain intensity, location, and quality; detailed neurological assessment, QST, and where appropriate specialised clinical investigations such as skin biopsy and nerve conduction studies (Tables 1 and 2 of Supplementary Methods, available at http://links.lww.com/PAIN/C169).

Participants recruited to all the cohorts were included based on their diagnosis of neuropathy and neuropathic pain. This included common causes of peripheral neuropathic pain, such as diabetic distal symmetrical polyneuropathy, as well as rare, extreme neuropathic pain disorders like erythromelalgia. Including rare, extreme cases of neuropathic pain offers the potential to uncover new mechanisms relevant to more common forms of neuropathic pain.^58^ Some of these conditions, such as IEM, may have a monogenic basis. However, most erythromelalgia cases are acquired, often associated with neuropathy, and were thus included in our analysis of painful and painless neuropathies. Participants with congenital insensitivity to pain, because of their distinct phenotype and significant loss of pain perception, were excluded from the broader neuropathic pain cohort when comparing painful and painless cases. Nevertheless, gene level rare variant analysis undertaken in participants with congenital insensitivity to pain did not identify any significant associations.

The clinical case definitions for each disorder are shown in Supplementary Table 2 (available at http://links.lww.com/PAIN/C169). Neuropathic pain was graded according to the NeupSIG grading system.^23^ A detailed description of the respective cohorts can be found in Supplementary Table 1 and Supplementary Methods (available at http://links.lww.com/PAIN/C169).

The inclusion in genetic analysis required the availability of DNA (or a blood sample from which DNA could be extracted), adequate clinical information for assessment of neuropathy and neuropathic pain grading according to the harmonised phenotyping protocol, informed consent from study participants for the use of their data and DNA, and ideally a measure of pain intensity (although this was not always available). The Brief Pain Inventory^13^ average pain score was the preferred measure for pain intensity. Brief Pain Inventory is a tool that offers several advantages: it is validated and reliable across different causes of neuropathic and chronic pain; it offers a comprehensive and multidimensional approach to measure pain because it includes items that assess pain at “worst, least, average and right now,” thus capturing variability over time; it is straightforward to administer; it ensures consistency with previous cohorts recruited, such as the PiNS cohort.^59^ If the Brief Pain Inventory average pain intensity score (this measure was used in DOLORisk but not all additional cohorts) was not available, for those participants with neuropathic pain, a pain score was imputed (see Supplementary Methods, available at http://links.lww.com/PAIN/C169).

### 2.2. Quantitative sensory testing

Quantitative sensory testing is a standardised psychophysical tool to assess somatosensory phenotypes. The anatomical area of neuropathic pain or neuropathy was tested with a modified DFNS (German research network of neuropathic pain) protocol.^37^ This protocol assesses 13 parameters, including thermal and mechanical detection, and pain thresholds. Raw QST data were normalised for age, sex and body site to generate *z*-scores. The *z*-score was subsequently used for downstream analysis. Certain modifications to QST protocol were made to improve efficiency. Specifically, mechanical pain sensitivity assessment was shortened from 5 to 2 rounds of tests; wind up ratio was performed in cases where it was helpful to include a measure of central sensitisation, such as in conditioned pain modulation; and thermal sensory limen was not performed in those with traumatic nerve injury. Participants were categorised into different somatosensory phenotype groups based on their QST results. This was done using 2 established algorithms, as each approach generates distinct groups:

(1) Unbiased cluster analysis identifies 3 distinct phenotypes: sensory loss, mechanical hyperalgesia, and thermal hyperalgesia.^5^ The assignment of individuals into these phenotypic groups is undertaken using the published deterministic algorithm.^66^ In this study, we combine participants from the thermal and mechanical hyperalgesia groups to increase statistical power. In polyneuropathy, where sensory loss predominates,^66^ merging the hyperalgesia groups facilitates a comparison between enhanced pain perception and sensory loss.
(2) Categorisation into irritable (characterised by conserved sensation and heightened sensitivity to pinprick stimuli) and nonirritable phenotype (the remainder).^16^

### 2.3. Genetic analyses

#### 2.3.1. Genome-wide association study genotyping

A total of 2740 samples were genotyped using the ChipArray Infinium Global Screening Array-24 v.2.0/v3.0 assay Infinium HTS and run on Illumina Iscan system. Standard quality control steps were applied.^2^ Genotype imputation was performed using the Michigan Imputation Server, with the European population serving as the reference (panel HRC r1.1 2016). Individuals of European ancestry and unrelated to the second degree were kept, and single-nucleotide polymorphisms (SNPs) with *R*^2^ < 0.4 and minor allele frequency <0.01 were filtered out (Supplementary Fig. 1, available at http://links.lww.com/PAIN/C169). A final dataset consisting of 7,837,857 SNPs and 2467 individuals with available phenotypic information was obtained for subsequent analyses.

We tested association of each SNP after imputation with various outcomes using regenie v3.3, a software for 2-step whole-genome regression modelling,^39^ under an additive model, with adjustment for age, sex, the first 10 principal components (PCs), batch/array version, and in some cases Toronto Clinical Scoring System (TCSS) as a measure of neuropathy severity was also used as covariant. We considered TCSS as a potential covariate in our modelling because it may be associated with the presence of common and rare variants and also have an impact on both the presence and the severity of neuropathic pain. Continuous phenotypic traits, including both primary outcomes and covariates were transformed using the rank-based inverse normal transformation (INT) to meet the assumption of normally distributed residuals in model fitting. We performed analyses for the following phenotypic outcomes: (1) neuropathic pain vs no neuropathic pain as a binary outcome, (2) neuropathic pain intensity as a quantitative variable (INT transformed), and (3) QST profiles designated as binary outcomes: “irritable nociceptor vs non-irritable nociceptor” and “sensory loss vs hyperalgesia.” In the context of peripheral neuropathy, we only included participants with probable and definite neuropathic pain according to the NeuPSIG criteria, but in the context of paroxysmal disorders, such as erythromelalgia, we included some individuals that fell into the category of possible neuropathic pain (as there may not be clinical sensory signs between attacks meaning that would not meet criteria for probable neuropathic pain).^23^ All analyses were performed in the entire cohort and separately in the participants with diabetic polyneuropathy.

#### 2.3.2. Whole-exome sequencing

To investigate the association of rare genomic variants and neuropathic pain, we performed WES on DNA from 1702 DOLORisk participants. Exome sequencing was performed in the Wellcome Centre for Human Genetics using the Twist Human Core Exome EF Multiplex Complete Kit as the basis, but with additional spiked-in probes to maximise capture of the 45 DOLORisk target genes (Supplementary Table 3, available at http://links.lww.com/PAIN/C169). These were selected (in 2017) on the basis of including those genes that had been associated with Mendelian human pain disorders, and additional gene selection was informed by a systematic review and meta-analysis of genetic risk factors for neuropathic pain and data from experimental studies in model organisms (Supplementary Table 3, available at http://links.lww.com/PAIN/C169).^64^

After quality control and preprocessing, we tested for associations with painful vs painless neuropathy. In each case, we used sex, age, PC 1-4, and batch as covariates (model 1), and when looking at diabetic participants only, we also considered sex, age, PC 1-4, TCSS for diabetic neuropathy and batch as covariates (model 2). Continuous phenotypic traits were INT transformed as for the GWAS above. After removing samples with missing covariates, we considered 1458 participants (1026 cases and 432 control subjects), of which 1048 were diabetic neuropathy cohort. Variants were annotated using VEP ensemble,^30^ and allele frequencies in the general population were obtained from GNOMAD v.2.2.1.^40^ Variant masks considered ClinVar, VEP impact, SIFT, PolyPhen, LOFTEE, and allele frequencies in the cohort and in the general population.

Group-wise associations were tested for rare variants on a subset of the 45 DOLORisk target genes that carried rare variants. Ten of 45 genes had rare variants survived filtering: *SPTLC1*, *PIEZO2*, *NTRK1*, *MMP1*, *TRPM8*, *HCN3*, *OPRM1*, *SCN3A*, *SCN9A*, and *SCN10A*. We tested for gene-wise associations using a variant component optimal test (SKAT-O) and reverse regression with a Wilcoxon test.^31^ Variants were named after the HGVS convention, and amino acids were numbered using both the ENSEMBL (1-based) and the UCSC (0-based) coordinate systems. A full description of genotyping, GWAS, and WES analysis can be found in Supplementary Methods (available at http://links.lww.com/PAIN/C169).

#### 2.3.3. Gene-based test, pathway, and enrichment analyses

For the gene-based test, pathway exploration, and enrichment analyses, we used the Functional Mapping and Annotation of Genome-Wide Association Studies (FUMA) software.^70^ This tool uses GWAS summary statistics as input to facilitate gene prioritisation, gene expression assessment, and pathway process enrichment. To mitigate the impact of multiple testing, FUMA applied the Bonferroni correction (*P*_bon_ < 0.05). FUMA also implements MAGMA gene-based and gene-set analysis.

#### 2.3.4. Phenome-wide association analysis

To examine potential associations between the top SNP associations and their corresponding genes identified in our present analyses and other related traits, we conducted a phenome-wide association analysis (PheWAS). This analysis involved a generation of PheWAS plots using an extensive dataset of 4756 GWAS summary statistics available on the GWAS ATLAS platform.^69^ Inclusion criteria encompassed all GWASs and their corresponding genes. For the PheWAS SNP plot, SNPs with *P* values <0.05 were considered. Adjustment for multiple comparisons was done using the Bonferroni correction method. In addition, we used Open Targets Genetics resources, which combine data from human GWAS and functional genomics, encompassing gene expression, protein levels, chromatin interactions, and conformation data across various cell types and tissues.^26^ This approach allows us to further confirm the connections between GWAS-associated loci, variants, and their probable causal genes.

#### 2.3.5. Polygenic risk scores

To understand the causal relationships between related traits (such as those relevant to glycaemia, lipid, inflammation, and mental health), we generated polygenic risk score (PRS) (using PRS-CS) for those traits and tested whether they were predictive of our various neuropathic pain phenotypes. PRS-CS was developed to deduce posterior SNP effect sizes using the principles of continuous shrinkage (CS) priors and was used to leverage GWAS summary statistics in conjunction with the 1000 Genomes Project phase 3 European samples as an external LD reference panel.^25^

We obtained summary statistics for a number of complex traits that could relate to neuropathy and pain including: glycaemic and metabolic traits, inflammation traits, major psychiatric disorders, and sleep traits. These summary statistics were sourced from publicly accessible repositories, namely the Psychiatric Genomics Consortium and the GWAS ATLAS.

### 2.4. Ethical approvals

All participants provided written informed consent in accordance with the Declaration of Helsinki. Details of the ethical approvals are given in Supplementary Methods (available at http://links.lww.com/PAIN/C169).

## 3. Results

### 3.1. Genome-wide association study, gene-based, pathway and enrichment analyses

**Figure 2.**
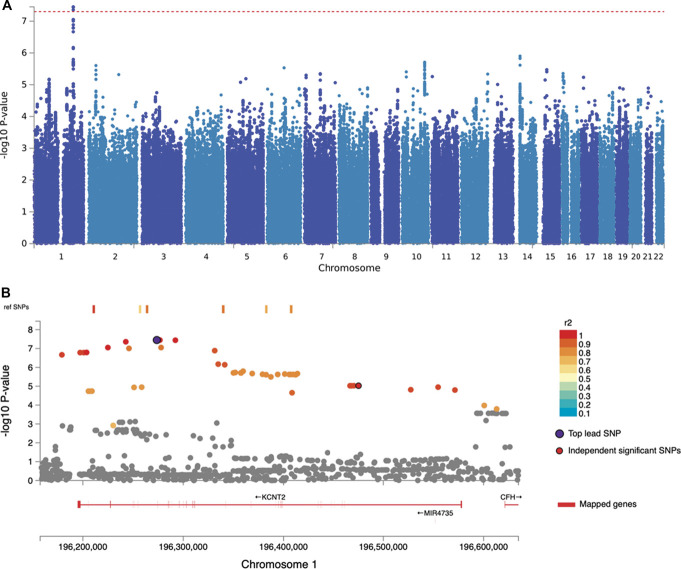
GWAS of neuropathic pain intensity in diabetic neuropathy cohort showed a significant SNP rs114159097. (A) Manhattan plot at the SNP-level, genome-wide significant level was highlighted by a horizontal red line at a threshold of 5 × 10^−8^. (B) Regional plot for the top lead SNP in the GWAS of neuropathic pain intensity in diabetic neuropathy. Each SNP is colour-coded based on the highest *r*^2^ to the top independent significant SNP. GWAS, genome-wide association study; SNP, single-nucleotide polymorphism.

The GWAS conducted on the entire cohort did not reveal association signals that reached the genome-wide significant level, either for neuropathic pain as a binary trait (n = 2186) or for pain intensity as a quantitative outcome (n = 2103) (Supplementary Figs. 2 and 3, available at http://links.lww.com/PAIN/C169). However, when focusing specifically on participants with diabetic neuropathy, the GWAS on neuropathic pain intensity (n = 1541) identified a genome-wide significant association with a locus located on chromosome 1q31.3 (Fig. 2, rs114159097, *P* = 3.55 × 10^−8^) within the potassium sodium–activated channel subfamily T member 2 (*KCNT2*) gene. Neuropathic pain is associated with neuropathy severity, and after additionally adjusting analyses for TCSS as a measure of neuropathy severity, the GWAS for binary (Supplementary Fig. 5A, available at http://links.lww.com/PAIN/C169) neuropathic pain vs no pain within diabetic cohort (n = 1028) showed a genome-wide significantly associated SNP (rs10919166, *P* = 1.07 × 10^−9^, see Table 1 for listed top SNPs) localised on the nitric oxide synthases 1 adaptor protein (*NOS1AP*) gene (Supplementary Fig. 5B, available at http://links.lww.com/PAIN/C169, see diabetic cohort binary measures GWAS in Supplementary Fig. 4, available at http://links.lww.com/PAIN/C169 and quantitative measures corrected for TCSS in Supplementary Fig. 6, available at http://links.lww.com/PAIN/C169, and top GWAS SNPs in Supplementary Table 4, available at http://links.lww.com/PAIN/C170).

**Table 1:** Top independent significant single-nucleotide polymorphisms from 4 genome-wide association studies.

GWAS	rsID	chr	A1	A2	MAF	*P*	β	Se	Nearest gene
GWAS for neuropathic pain intensity within diabetic neuropathy M1	**rs114159097**	1	C	G	0.026	**3.55 × 10** ^**−8**^	0.51	0.09	***KCNT2*** (potassium sodium-activated channel subfamily T member 2)
	rs62132430	2	C	G	0.077	2.49 × 10^−6^	0.24	0.05	AC009414.1
	rs112716392	6	A	T	0.005	2.94 × 10^−6^	0.63	0.14	RP11-33E24.3
	rs115197918	2	A	G	0.034	3.49 × 10^−6^	0.46	0.1	AC009414.1
	rs114485796	2	G	A	0.016	4.81 × 10^−6^	0.45	0.1	AC007364.1
GWAS for binary neuropathic pain vs no pain within diabetic cohort M2	**rs10919166**	1	A	G	0.268	**1.07 × 10** ^**−9**^	−0.77	0.13	***NOS1AP*** (nitric oxide synthase 1 adaptor protein)
	rs11196200	10	G	C	0.483	1.90 × 10^−7^	−0.59	0.11	*TCF7L2* (transcription factor 7 like 2)
	rs75532353	8	C	T	0.018	4.18 × 10^−7^	−2.15	0.46	*ASAP1* (ArfGAP with SH3 domain, ankyrin repeat and PH domain 1)
	rs73184435	3	A	G	0.081	4.33 × 10^−7^	−0.99	0.19	*TPRG1* (tumor protein P63 regulated 1)
	rs12185526	19	G	C	0.017	7.46 × 10^−7^	−1.87	0.39	*MYO9B* (myosin IXB)
GWAS for irritable nociceptor vs nonirritable nociceptor on the entire cohort M1	**rs72669682**	4	A	G	0.027	**4.39 × 10** ^**−8**^	1.88	0.32	***ANK2*** (ankyrin 2)
	rs12571618	10	A	G	0.076	7.30 × 10^−7^	1.15	0.22	RP11-482E14.1
	rs150499233	14	G	C	0.017	8.98 × 10^−7^	2.34	0.45	RP11-736N17.8
	rs150964961	4	A	T	0.009	1.57 × 10^−6^	2.49	0.5	RP11-148B6.2
	rs9820034	3	C	T	0.175	1.94 × 10^−6^	−1.02	0.23	*TNIK* (TRAF2 and NCK interacting kinase)
GWAS for sensory loss vs hyperalgesia on the entire cohort M2	**rs141853415**	17	A	G	0.022	**1.25 × 10** ^**−10**^	−4.25	0.99	**AC015815.5**
	rs10894219	11	A	G	0.201	1.64 × 10^−7^	0.75	0.14	*BAK1P2* (BCL2 antagonist/killer 1 pseudogene 2)
	rs28924101	1	G	A	0.104	1.25 × 10^−6^	0.8	0.17	*MAD2L2* (mitotic arrest deficient 2 like 2)
	rs17852649	1	G	T	0.388	1.57 × 10^−6^	0.57	0.12	*IL22RA1* (interleukin 22 receptor subunit alpha 1)
	rs2032567	6	T	C	0.291	1.92 × 10^−6^	−0.56	0.12	*LAMA4* (laminin subunit alpha 4)

M1: model 1 as co-variates include sex, age, batch, and principal components 1 to 10.

M2: model 2 as co-variates include sex, age, batch, and principal components 1 to 10 and TCSS (as a measure of neuropathy severity).

GWAS, genome-wide association study; MAF, minor allele frequency; TCSS, Toronto Clinical Scoring System.

The top lead GWAS results were highlighted in bold for clarity.

**Figure 3.**
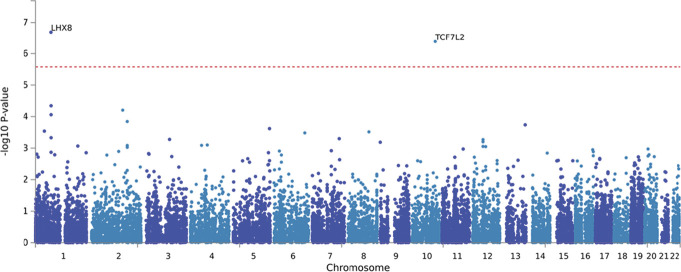
Manhattan plot (gene-based test) for binary measures of neuropathic pain vs no pain in diabetic neuropathy cohort revealed 2 significant genes. Input SNPs were mapped to 18,829 protein coding genes. Genome-wide significance (red dashed line in the plot) was defined at *P* = 0.05/18,829 = 2.655 × 10^−6^. SNP, single-nucleotide polymorphism.

The gene-based MAGMA analyses for binary measures of neuropathic pain vs no pain within the participants with diabetes revealed 2 significantly associated genes: Lim homeobox 8 (*LHX8*, *P* = 2.26 × 10^−7^) and transcription factor 7 like 2 (*TCF7L2*, *P* = 7.80 × 10^−7^) with a genome-wide significance threshold of *P* = 2.655 × 10^−6^ for 18,829 mapped protein coding genes (Fig. 3). In addition, *TCF7L2* also emerged as a significant gene in the MAGMA gene-based tests for the binary GWAS adjusting for TCSS in the diabetic cohort (*P* = 1.52 × 10^−6^) (Supplementary Fig. 7, available at http://links.lww.com/PAIN/C169).

### 3.2. Fine mapping and expression quantitative trait loci

rs114159097 is located within *KCNT2* gene (Fig. 2B). A subset of available SNPs showed linkage disequilibrium with rs114159097 (*R*^2^ > 0.6) mapping to the *KCNT2* region as depicted in Figure 2. Furthermore, rs114159097 was identified as a cis-expression quantitative trait locus (cis-eQTL) for complement factor H (*CFH*) gene (*z* = −4.77, *P* = 1.83 × 10^−6^) in the eQTLGen database and Open Targets Genetics (eQTL = 0.1, Supplementary Table 5, available at http://links.lww.com/PAIN/C170). Both *KCNT2* and *CFH* genes were mapped by chromatin interactions and eQTLs (Supplementary Fig. 8, available at http://links.lww.com/PAIN/C169). Regional plot shows that rs10919166 is located within the *NOS1AP* gene (Supplementary Fig. 5B, available at http://links.lww.com/PAIN/C169 regional plot) and serves as eQTLs for several genes (Supplementary Fig. 9, available at http://links.lww.com/PAIN/C169 for chromatin interaction), including *C1orf226* (eQTLGen and Open Targets Genetics, Supplementary Table 5, available at http://links.lww.com/PAIN/C170), *NOS1AP* (BIOSQTL & eQTLGen), *HSPA7* (GTEx, in tissue skin), and *FCGR2C* (GTEx, in tissue skin), according to various eQTL databases. Both *C1orf226* and *NOS1AP* genes were highlighted by chromatin interactions and eQTLs (mapped in red in Supplementary Fig. 9, available at http://links.lww.com/PAIN/C169).

### 3.3. Phenome-wide association analysis

Phenome-wide association analysis was conducted using the GWAS Atlas platform and Open Target Genetics to explore secondary phenotypes associated with rs114159097, rs10919166, *KCNT2*, *LHX8*, *NOS1AP*, and *TCF7L2* genes. rs114159097 was significantly associated with age-related macular degeneration trait (*P* = 5.43 × 10^−6^), and rs10919166 exhibited an association with neovascular disease (*P* = 5.65 × 10^−5^). *LHX8* was found to be associated with educational attainment (*P* = 9.75 × 10^−11^), educational qualifications (*P* = 5.88 × 10^−7^), and osteoarthritis (*P* = 9.06 × 10^−6^), and the *KCNT2* and *NOS1AP* genes displayed associations with 34 diverse traits after Bonferroni correction, encompassing ophthalmological, cellular, psychiatric, immunological, cardiovascular, and metabolic domains (refer to Supplementary Tables 6 and 7, available at http://links.lww.com/PAIN/C169). *TCF7L2* gene had a strong association with diabetes traits, with *P* values ranging from 2.26 × 10^−10^ to 1.49 × 10^−154^, including diabetes and type 2 diabetes. Other traits including metabolic traits, cardiovascular, haematological, and psychiatric domains also showed significant associations (see Supplementary Table 8, available at http://links.lww.com/PAIN/C170).

Moreover, Open Targets Genetics revealed that rs114159097 is negatively associated with various measurement traits including overall health rating and sleep change, positively associated with speech disorders (Supplementary Table 9, available at http://links.lww.com/PAIN/C170). rs10919166 is positively associated with traits in cell proliferation disorders and biological process (Supplementary Table 9, available at http://links.lww.com/PAIN/C170).

### 3.4. Polygenic risk score analysis

We examined whether PRS developed for glycaemic traits, inflammation traits, major psychiatric disorders, sleep, and lipid traits would be predictive of neuropathic pain status (both the binary outcome and quantitative measures) in individuals with diabetes and the entire cohort (Table 2). These analyses were performed using publicly available GWAS summary statistics downloaded from the PGC and the GWAS ATLAS. The PRS for depression showed a consistent positive association with neuropathic pain (*P*_binary_ and *P*_quantitative_ = 0.01) in the entire cohort. Polygenic risk score for C-reactive protein (CRP, as a marker of inflammation) was positively associated (*P*_binary_ and *P*_quantitative_ = 0.01) with neuropathic pain in diabetic polyneuropathy. The fasting insulin PRS displayed a negative association with neuropathic pain in the whole cohort and in diabetic polyneuropathy subgroup (*P*_binary_ = 0.04, and *P*_binary_ = 0.01).

**Table 2:** Glycaemic, inflammation, psychiatric, sleep, and lipids-related traits polygenic risk scores analysis.

Traits	Diabetic quantitative			Top 30%			Diabetic binary			Top 30%			Whole binary			Whole quantitative		
	Estimate	*R* ^2^	*P*	Estimate	*R* ^2^	*P*	Estimate	*R* ^2^	*P*	Estimate	*R* ^2^	*P*	Estimate	*R* ^2^	*P*	Estimate	*R* ^2^	*P*
Fasting glucose	−1.24E+05	0.00%	0.19	−4.70E+04	0.14%	0.12	−1.24E+05	0.16%	0.17	−4.42E+04	0.27%	0.08	−1.33E+05	0.19%	0.08	−1.35E+05	0.09%	0.16
2-h glucose after an oral glucose challenge	−8.13E+04	0.00%	0.78	−5.47E+04	0.05%	0.36	−1.80E+05	0.04%	0.50	−4.53E+04	0.07%	0.36	−2.28E+05	0.06%	0.31	−1.36E+05	0.01%	0.64
**Fasting insulin**	−3.96E+05	0.17%	0.10	−4.32E+04	0.04%	0.39	**−4.86E+05**	**0.55%**	**0.01★**	**−8.36E+04**	**0.36%**	**0.04★**	**−3.37E+05**	**0.27%**	**0.04★**	−3.05E+05	0.10%	0.14
HbA1c	−1.34E+05	0.00%	0.19	−1.79E+04	0.02%	0.58	−6.19E+04	0.04%	0.52	−1.93E+04	0.05%	0.46	−4.13E+04	0.02%	0.61	−1.41E+05	0.08%	0.17
C-reactive protein	**2.07E+05**	**0.41%**	**0.01★**	**8.06E+04**	**0.77%**	**0.0004★**	8.88E+04	0.16%	0.17	**4.77E+04**	**0.57%**	**0.01★**	3.26E+04	0.02%	0.55	9.25E+04	0.08%	0.19
Schizophrenia	4.99E+04	0.03%	0.53	4.28E+02	0.00%	0.98	1.48E+04	0.00%	0.82	6.56E+03	0.02%	0.66	3.87E+04	0.03%	0.47	3.87E+04	2.05%	0.47
Alcohol use disorder	2.46E+05	0.05%	0.37	**8.32E+04**	**0.36%**	**0.016★**	−1.62E+05	0.05%	0.46	5.04E+04	0.28%	0.07	−1.24E+05	0.03%	0.50	1.51E+05	0.02%	0.53
Bipolar disorder	2.09E+05	0.04%	0.43	1.30E+04	0.01%	0.79	−1.35E+05	0.03%	0.52	1.37E+04	0.01%	0.72	−1.61E+05	0.05%	0.37	1.04E+05	0.01%	0.65
Panic disorder	−4.29E+04	0.01%	0.78	1.06E+04	0.03%	0.52	5.50E+04	0.02%	0.67	8.91E+03	0.04%	0.50	1.24E+05	0.08%	0.25	1.24E+05	2.11%	0.25
Anxiety disorders	−4.91E+05	0.21%	0.06	−5.89E+04	0.20%	0.07	−3.31E+05	0.20%	0.12	−3.31E+05	0.20%	0.12	−1.94E+05	0.07%	0.28	−2.93E+05	0.07%	0.20
Eating disorder (anorexia nervosa)	9.94E+04	0.02%	0.62	1.20E+04	0.01%	0.63	1.28E+05	0.05%	0.43	1.92E+03	0.00%	0.92	1.37E+05	0.06%	0.32	−1.70E+04	0.00%	0.92
Depression	**1.09E+06**	**0.46%**	**0.01★**	9.25E+04	0.16%	0.10	**9.17E+05**	**0.69%**	**0.005★**	**9.57E+04**	**0.37%**	**0.04★**	**7.10E+05**	**0.41%**	**0.01★**	**8.86E+05**	**0.28%**	**0.01★**
Self-reported daytime sleepiness	−1.76E+04	0.00%	0.97	−1.81E+04	0.01%	0.78	1.40E+05	0.01%	0.69	−2.05E+04	0.01%	0.69	3.95E+04	0.00%	0.89	−7.54E+04	0.00%	0.84
Insomnia symptoms	4.81E+05	0.11%	0.17	7.40E+04	0.12%	0.16	4.95E+05	0.26%	0.08	5.12E+04	0.12%	0.23	**5.81E+05**	**0.36%**	**0.02★**	5.50E+05	0.14%	0.08
Overall sleep duration	−3.13E+04	0.00%	0.91	−1.29E+04	0.01%	0.80	−1.42E+05	0.04%	0.51	−5.43E+03	0.00%	0.89	−2.78E+05	0.14%	0.13	−4.45E+04	0.00%	0.85
Long sleep duration	2.16E+05	0.09%	0.24	3.09E+04	0.11%	0.17	−1.55E+03	0.00%	0.99	1.82E+04	0.08%	0.33	3.83E+04	0.01%	0.76	2.37E+05	0.09%	0.15
Short sleep duration	1.31E+05	0.03%	0.52	3.56E+04	0.09%	0.22	1.39E+05	0.06%	0.40	4.56E+03	0.00%	0.85	2.05E+05	0.13%	0.14	2.39E+05	0.08%	0.17
High-density lipoprotein cholesterol	−1.10E+05	0.13%	0.14	−6.09E+04	0.24%	0.06	−9.15E+04	0.19%	0.14	−4.28E+04	0.26%	0.08	4.90E+03	0.00%	0.92	3.34E+04	0.01%	0.61
Non–high-density lipoprotein cholesterol	5.33E+04	0.02%	0.56	2.64E+04	0.04%	0.40	8.66E+04	0.12%	0.24	2.57E+04	0.09%	0.32	5.54E-06	0.10%	0.20	1.02E+05	0.08%	0.19
Low-density lipoprotein cholesterol	1.87E+04	0.00%	0.83	1.57E+04	0.02%	0.63	5.06E+04	0.04%	0.47	1.67E+04	0.04%	0.50	7.59E+04	0.10%	0.19	9.28E+04	0.07%	0.22
Total cholesterol	6.05E+03	1.70%	0.94	2.70E+03	0.00%	0.93	1.02E+04	0.00%	0.87	1.24E+03	0.00%	0.96	5.00E+04	0.06%	0.33	9.62E+04	0.10%	0.14
Triglycerides	2.93E+04	0.01%	0.72	2.74E+04	0.05%	0.39	4.83E+03	0.00%	0.94	2.56E+03	0.00%	0.92	−2.94E+04	0.02%	0.59	−8.64E+03	0.00%	0.90

Diabetic quantitative: quantitative measures of neuropathic pain in the diabetic polyneuropathy cohort.

Diabetic binary: binary measures of neuropathic pain in the diabetic polyneuropathy cohort.

Whole quantitative: quantitative measures of neuropathic pain in the whole cohort.

Whole binary: binary measures of neuropathic pain in the whole cohort.

Top 30%: genetic variants that had *P* values ranged from the top 30% of GWAS summary statistics.

GWAS, genome-wide association study.

PRS results with p < 0.05 were highlighted in bold with an asterisk (*) to indicate statistical significance.

### 3.5. Whole exome sequencing and candidate gene analysis

We tested the hypothesis that groups of rare variants on the 45 DOLORisk target genes might have a collective effect associated with the presence or absence of neuropathic pain. When looking at the whole gene set, we found no exome-wide significant association. After filtering for nonsynonymous variants that were not common in our cohort, minor allele frequency <0.05, and rare in the general population, GNOMAD Non-Finnish Europeans AF <0.01, and also filtering according to the predicted impact of the variant on the encoded protein, we were able to test for associations in groups of variants found in 10 of our DOLORisk target genes. We tested for association between these 10 genes and the presence of neuropathic pain in the whole cohort and in diabetic participants only, adjusting for the following covariates: sex, age, batch, and PCs 1 to 4 (model 1) or additionally including TCSS (as a measure of neuropathy severity, model 2). We found Bonferroni-corrected significant associations for the number of genes tested, with groups of variants in *OPRM1* (4 variants, reverse regression *P* = 0.0037, diabetics only, model 2) and *SCN9A* (minAC = 1: 12, minAC = 3: 6 variants, EMMAX burden test *P* = 0.0037, β = 0.33, burden count = 73, whole cohort, model 1 and reverse regression *P* = 0.0033, diabetics only, model 1) genes, Supplementary Table 10 (available at http://links.lww.com/PAIN/C169).

**Figure 4.**
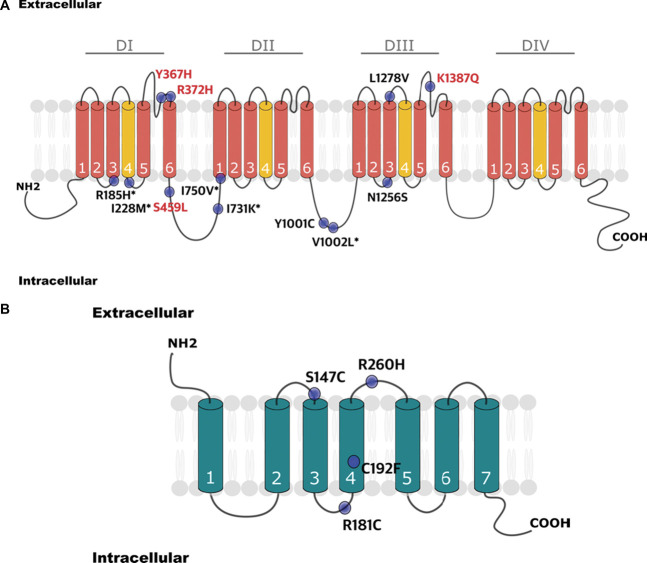
Protein structure pattern of the *SCN9A* and *OPRM1* genes showing localisation of the rare variants within each channel. (A) The schematic representation of the human voltage-gated sodium channel Nav1.7 alpha-subunit (encoded by the *SCN9A* gene) shows novel *SCN9A* variants (not previously reported in the literature) in red, and gain of function mutations previously characterised in black (*). The channel consists of 4 domains, each comprising 6 transmembrane segments (1-6). Within each domain, the loops between the transmembrane segments 5 and 6 constitute the ion selectivity filter, whereas the voltage sensor domain is located in transmembrane segment 4. (B) Human *OPRM1* (mu opioid receptor) channel, with variants depicted in black. Variants pathogenicity is reported according to ClinVar and functional studies, as shown in Supplementary Tables 11 and 12 (available at http://links.lww.com/PAIN/C169).

These associations were driven by novel and known variants that were more frequent in the painful vs the painless participants. This is consistent with a gain of function of *SCN9A*, which encodes the voltage-gated sodium channel Na_V_1.7. Indeed 5 of these 12 variants have previously been linked to neuropathic pain and have been shown to cause gain of function on electrophysiological analysis (detailed in Supplementary Table 11, available at http://links.lww.com/PAIN/C169, Fig. 4A). *OPRM1* encodes the mu opioid receptor (MOR) all variants were more frequent in painful vs painless participants, none had previously been associated with neuropathic pain but interestingly these variants have previously been shown to alter MOR signalling (detailed in Supplementary Table 12, available at http://links.lww.com/PAIN/C169, Fig. 4B).

### 3.6. Quantitative sensory testing analysis

**Figure 5.**
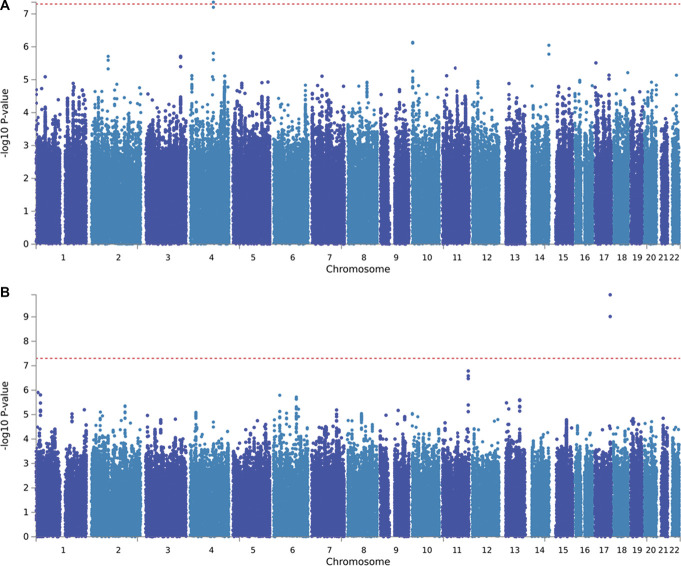
QST irritable nociceptor vs nonirritable nociceptor phenotype GWAS and sensory loss vs hyperalgesia phenotype in the whole cohort. (A) Irritable nociceptor vs nonirritable nociceptor QST phenotype GWAS in the whole cohort Manhattan plot at the SNP-level, genome-wide significant level was highlighted by a horizontal red line at a threshold of 5 × 10^−8^. (B) Sensory loss vs hyperalgesia QST phenotype GWAS corrected for TCSS in the whole cohort Manhattan plot. GWAS, genome-wide association study; QST, quantitative sensory testing; SNP, single-nucleotide polymorphism; TCSS, Toronto Clinical Scoring System.

We also performed a GWAS on sensory profiles defined using QST. We conducted a GWAS on the entire cohort, where we classified participants into 2 groups: those with irritable nociceptor (n = 170) phenotype and those with nonirritable nociceptor (n = 916) QST phenotypes. We identified 1 SNP with genome-wide significance, rs72669682 (4.39 × 10^−8^), located on chromosome 4q25-q26 within the gene *ANK2* (Fig. 5A). In full cohort sensory loss (n = 526) vs hyperalgesia (n = 380) adjusting for TCSS GWAS, we also identified a significant SNP rs141853415 (1.25 × 10^−10^, Fig. 5B). Furthermore, because we had found the SNP rs114159097 (within the *KCNT2* gene) was significantly associated with neuropathic pain intensity in the diabetic neuropathy individuals, we next checked if diabetic individuals carrying SNP rs114159097 exhibited distinct QST sensory profiles compared with diabetic participants without this SNP. The results indicate that there were differences in the mechanical pain sensitivity (*P* = 0.0001) with enhanced mechanical pain sensitivity in those individuals with the minor allele (Supplementary Fig. 12, available at http://links.lww.com/PAIN/C169).

Analysing gene-level associations of groups of rare variants identified through targeted WES in individuals with irritable nociceptors vs nonirritable nociceptors, we identified 1 variant with significance exceeding *P* < 5 × 10^−5^. The most noteworthy variants were situated near the *NBPF13P* gene (rs113126882, odds ratio = 0.27, *P* = 5.79 × 10^−6^, Supplementary Fig. 10, available at http://links.lww.com/PAIN/C169). Similarly, examining gene-level associations of rare variants in individuals with sensory loss vs hyperalgesia revealed 1 variant with significance exceeding *P* < 5 × 10^−5^, with the top variant located near the *ZNF679* gene (Supplementary Fig. 11, available at http://links.lww.com/PAIN/C169, rs10949885, odds ratio = 0.57, *P* = 1.31 × 10^−6^).

## 4. Discussion

DOLORisk implemented a protocol,^63^ designed to accurately define neuropathic pain.^23^ In our GWAS analyses, we found significant association of neuropathic pain mapping to the *KCNT2* locus in diabetic neuropathy. Individuals carrying *KCNT2* SNP rs114159097 exhibited a distinct QST sensory profile with enhanced mechanical pain sensitivity. Polygenic risk score analysis for traits relevant to diabetes found that C-reactive protein showed a positive association, whereas PRS for fasting insulin showed a negative association with neuropathic pain, in individuals with diabetic polyneuropathy. A candidate gene analysis identified a number of rare variants in DOLORisk priority genes *OPRM1* and *SCN9a* enriched in the neuropathic pain group. Finally, a more exploratory GWAS of QST-defined sensory phenotypes identified SNPs within the *ANK2* gene and an intergenic variant (rs141853415) on chromosome 17.

The locus that showed the strongest association with severity of diabetic polyneuropathy-related pain mapped to the gene *KCNT2*. *KCNT1* and *KCNT2* encode 2 homologous potassium channels activated by cytosolic Na^+^ (KNa),^27,38^ which are broadly expressed in the CNS and by sensory neurons. This gene has not previously been linked to human pain^41^; however, preclinical studies have implicated *KCNT2* in neuropathic pain.^60^ When using neuropathy severity as a covariate, we found a significant association between the locus nitric oxide synthase-1 adaptor protein (*NOS1AP*, lead SNP rs10919166) and painful diabetic neuropathy. NOS1AP interacts with neuronal NOS in a manner that can modulate glutamatergic signalling and has been linked to neuropathic pain in mouse models.^32^

We also saw a signal at gene level with *LHX8* and *TCF7L2* and neuropathic pain in diabetic neuropathy. *LHX8* belongs to the LIM-Homeobox family and is essential for the development of cholinergic neurons in the forebrain^32,46^; these neurons have recently been shown to suppress pathological pain.^46^ The specific role of *LHX8* in preclinical pain models has yet to be investigated. *TCF7L2* has been identified as the strongest risk locus for type 2 diabetes in multiple populations.^15^ *TCF7L2* has also been reported to alter brain function but has not previously been linked to pain.^17^

The construction of PRSs using GWAS data can help in elucidation of the genetic interconnections among diverse traits. The associations observed with CRP, alcohol use disorder, depression, and insomnia symptoms support potential common genetic factors underpinning these traits and neuropathic pain. Epidemiological studies have shown an association between chronic musculoskeletal pain and insomnia and a complex interplay with CRP.^53^ C-reactive protein is an acute inflammatory protein that can increase significantly in response to infection or inflammation^57^ and has been implicated in both depression and neuropathic pain.^36,62^

The gene-burden analysis of rare variants in our 45 target pain genes revealed significant associations with neuropathic pain in diabetic neuropathy for 2 genes: *OPRM1* and *SCN9A*. *OPRM1* encodes the MOR, which has a key role in endogenous pain modulation.^56^ A recent meta-analysis examining the common variant in *OPRM1* (c.A118G; p.Asn40Asp) did not find significant association with neuropathic pain.^64^ However, we found 4 rare *OPRM1* variants overrepresented in the painful diabetic neuropathy group; these have previously undergone investigation for their impact on MOR signaling. The p.Arg260His missense variant, impaired basal G-protein coupling of MOR,^68^ and the p.Arg181Cys variant have severe functional impact resulting in “signaling dead” MOR.^49^ Heterozygous carriers of p.Arg181Cys require higher doses of morphine to elicit pain relief and homozygotes have markedly impaired response to morphine.^54^ The p.Ser147Cys and p.Cys192Phe variants were not found to alter signalling coupling or internalisation of MOR but could shift morphine potency (a large rightward shift in the case of p.Cys192Phe and more subtle leftward shift in the case of p.Ser147Cys). The fact that variants that impact on MOR are overrepresented in individuals who develop neuropathic pain after diabetic neuropathy aligns with the hypothesis that altered endogenous pain modulation may have a role in the development of neuropathic pain.^52^

Variants in *SCN9A* that encode the voltage-gated sodium channel Na_v_1.7 have previously been linked to multiple pain disorders, including the Mendelian extreme pain disorders IEM^73^ and PEPD^21^ as well as a risk factor for the more prevalent small fibre neuropathy.^19^ A number of these *SCN9A* variants that were driving the association with painful diabetic neuropathy have previously had functional analysis supporting gain of function. The p.Ile731Lys, p.Ile750Val, and p.Ile228Met variants have all previously been associated with small fibre neuropathy and shown to impair slow inactivation of Na_v_1.7 leading to dorsal root ganglion (DRG) neuron hyperexcitability.^19,28^ The p.Ile228Met variant led to impaired axon outgrowth when expressed in rodent DRG neurons.^19,48^ Young Ile228Met mutant mice demonstrated DRG neuron hyperexcitability^12^ followed by a paradoxical hypo-excitability phenotype in aged mice.^71^ In the zebrafish animal model, knock-in of this variant produces loss of small fibres and increase in temperature-dependent activity.^18^

p.Val1002Leu is a variant derived from Neanderthal introgression^74^ and is associated with gain of function in the form of resurgent currents and hyperexcitability when expressed in rodent DRG neurons.^19^ It was originally described in the context of small fibre neuropathy but is present at population level in healthy individuals and is linked to mechanical pain hypersensitivity.^20^

The p.Arg185His variant has previously been reported in small fibre neuropathy,^28^ painful diabetic neuropathy,^10^ and nonfreezing cold injury.^58^ Arg185His has been found to impair inactivation of Na_V_1.7 at cool temperatures^58^ and increase resurgent currents when transfected into rodent DRG neurons.^28^ Mice carrying the Arg185His variant showed enhanced evoked and spontaneous pain-related behaviour.^72^ The gain of function effects of the variants associated with the painful diabetic neuropathy in our cohort were more subtle than the striking changes in channel function caused by variants causing IEM and PEPD^7^; they probably act as a risk factor that only manifests as clinical neuropathic pain after interaction with other factors such as the metabolic derangement of diabetes or psychosocial factors.

Previous studies on *SCN9A* and painful diabetic neuropathy have reported some conflicting findings. Some studies found a higher frequency of rare *SCN9a* variants in painful compared with painless diabetic neuropathy (including variants with gain of function characteristics).^10,34^ A recent study by the PROPANE group reported potentially pathogenic variants in *SCN9A* in a similar proportion in painful and painless diabetic neuropathy (3.0% and 2.9% of participants, respectively), although no formal gene burden analysis was conducted.^1^ These discrepancies may be related to differences in both case definition and selection, variant filtering, and analytic tools deployed.

A substantial proportion of our participants underwent sensory profiling using QST. Distinct patterns of sensory profile can be found across large samples of patients with different aetiologies of neuropathic pain; these likely reflect underlying pathophysiological mechanisms.^1,5^ One method of dichotomising these profiles is the “irritable nociceptor” profile, in which small-fibre function is relatively preserved and associated with hyperalgesia and a “deafferentation profile” dominated by sensory loss.^22^ The “irritable nociceptor” profile was hypothesised to be associated with increased activity of nociceptors and indeed was predictive of a better response to oxcarbazepine, a drug which blocks sodium channels.^16^ In our GWAS comparing the “irritable” nociceptor with the “nonirritable nociceptor” group, the most significant SNP, rs72669682, was found within the *ANK2* gene. The *ANK2* gene is responsible for encoding a protein within the ankyrin family crucial for positioning and stabilising ion transporters and channels in the membranes of neurons.^44^

## 5. Strengths and limitations

The present study has several strengths: it includes the largest multicentre cohort to date of participants with deeply-phenotyped and harmonised information on neuropathic pain; it integrates a combination of discovery GWAS and candidate gene WES approaches; the deep phenotyping approach enabled us to focus on participants with probable and definite neuropathic pain and optimise a control group with painless neuropathy. A limitation is selection bias related to neuropathic pain assessments in secondary care; this was mitigated by inclusion of multiple centres including primary care networks in participant identification. The cohort sample size is still limited and our findings will require replication. National level biobanks such as UK-Biobank^6^ or the Million Veterans Program^24^ provide clinical and genetic data on large numbers of participants. Such cohorts can assess pain intensity in hundreds of thousands of individuals; however, the Million Veterans Program did not assess pain subtypes, and even when using dedicated screening questionnaires for neuropathic pain, these only allow the grading of “possible” neuropathic pain.^23,63^ With the technology and resources available to us at the time, the selection of target genes for analysis of rare variants was not comprehensive, and we were not powered to directly compare distinct aetiologies. Finally, we used the conventional threshold *P* < 5 × 10^−8^ to determine genome-wide significance. We tested 3 hypotheses that we considered independent. We did test 2 different models of association within each hypothesis, and we acknowledge that this might have an effect in the inflation of type I errors but only in a subset of the diabetic cohort.

In conclusion, DOLORisk has shown the advantages of a harmonised deep phenotyping approach in relation to consistency and the ability to illuminate insights into genetic associations of multiple neuropathic pain outcomes including pain report and sensory profile.

## Conflict of interest statement

D. L. Bennett has acted as a consultant in the last 2 years for AditumBio, Amgen, Biogen, Biointervene, Combigene, LatigoBio, GSK, Ionis, Lexicon therapeutics, Lilly, Neuvati, Novo Ventures, Orion, Replay, SC Health Managers, Third Rock ventures, Vida Ventures on behalf of Oxford University Innovation. He has received research funding from Eli Lilly and Astra Zeneca. He has received an industrial partnership grant from the BBSRC and AstraZeneca. B. Smith has received research funding from Eli Lilly. N. Attal has received consultancy fees or participated as speaker bureau in the last 2 years for Merz, Grunenthal, Biogen, Novartis, Medtronic, Pfizer and Viatris outside the submitted work. N. B. Finnerup has acted as consultant for PharmNovo, Vertex, NeuroPN, Saniona, Nanobiotix, Neurvati, Biogen, Merz, and Confo Therapeutics. She has received grants from IMI2PainCare an EU IMI 2 (Innovative medicines initiative) public–private consortium, and the companies involved are: Grunenthal, Bayer, Eli Lilly, Esteve, and Teva, outside the submitted work. D. Bouhassira has received consultancy fees from Grunenthal and Bayer in the last 2 years. N. van Zuydam is currently an employee of AstraZeneca and a shareholder of AstraZeneca stock. R. Baron is supported by the EUROPAIN project, which is a public–private partnership and has received support from the Innovative Medicines Initiative Joint Undertaking under grant agreement no. 115007, resources for which are composed of financial contribution from the European Union's Seventh Framework Programme (FP7/2007-2013) and European Federation of Pharmaceutical Industries and Associations (EFPIA) companies' in-kind contribution. The NEUROPAIN project is an investigator-initiated European multicentre study with R. Baron as the principal investigator and 10 co-investigator sites, supported by an independent investigator-initiated research grant from Pfizer Ltd. R. Baron has also received Research grant funding from: Pfizer Pharma GmbH, Grünenthal GmbH, Mundipharma Research GmbH und Co. KG., Alnylam Pharmaceuticals Inc., Zambon GmbH, Sanofi Aventis GmbH, Viatris. The funding source had no role in study design, data collection and analysis, or writing of the manuscript. Outside the submitted word, J. Gierthmühlen has received consultancy fees from TEVA and Omega Pharma. She has received grants from companies: ElectroZeutica, Bosana GmBh, and Neurotech GmbH and personal fees for lectures from Teva, Abbvie, Lilly GmbH, Lundbeck, Grünenthal, CHangePain, StreamUp, MediSage, CampusWebinar. A. S.C. Rice interests occurring in last 24 months: Officer (President-Elect) of International Association for the Study of Pain; ASCR undertakes consultancy and advisory board work for Imperial College Consultants in the last 24 months this has included remunerated work for: AstraZeneca, Pharmnovo, Confo and Combigene. A. S.C. Rice is named as an inventor on patents: Rice ASC, Vandevoorde S, Lambert D.M Methods using N-(2-propenyl) hexadecanamide and related amides to relieve pain. WO 2005/079771, Okuse K. et al. Methods of treating pain by inhibition of vgf activity EP13702262.0/WO2013 110945. Member Joint Committee on Vaccine and Immunisation- varicella sub-committee; Analgesic Clinical Trial Translation: Innovations, Opportunities, and Networks (ACTTION) steering committee member; Medicines and Healthcare products Regulatory Agency (MHRA), Commission on Human Medicines—Neurology, Pain & Psychiatry Expert Advisory Group. Grants and studentships—UKRI (Medical Research Council & BBSRC), Versus Arthritis, Alan and Sheila Diamond Trust, Royal British Legion, European Commission, Ministry of Defence, Dr. Jennie Gwynn Bequests, The British Pain Society, Royal Society of Medicine. R. Baron has acted as a consultant for Pfizer Pharma GmbH, Sanofi Aventis GmbH, Grünenthal GmbH, Lilly, Novartis Pharma GmbH, Bristol-Myers Squibb, Biogenidec, AstraZeneca GmbH, Daiichi Sankyo, Glenmark Pharmaceuticals S.A., Seqirus Australia Pty Ltd, Teva Pharmaceuticals Europe Niederlande, Teva GmbH, Genentech, Mundipharma International Ltd UK, Galapagos NV, Kyowa Kirin GmbH, Vertex Pharmaceuticals Inc, Biotest AG, Celgene GmbH, Desitin Arzneimittel GmbH, Regeneron Pharmaceuticals Inc USA, Theranexus DSV CEA Frankreich, Abbott Products Operations AG Schweiz, Bayer AG, Grünenthal Pharma AG Schweiz, Akcea Therapeutics Germany GmbH, Asahi Kasei Pharma Corporation, AbbVie Deutschland GmbH & Co KG, Air Liquide Sante International Frankreich, Alnylam Germany GmbH, Lateral Pharma Pty Ltd, Hexal AG, Angelini, Janssen, SIMR Biotech Pty Ltd Australien, Confo Therapeutics N. V. Belgium, Merz Pharmaceuticals GmbH, Neumentum Inc, F. Hoffmann-La Roche Ltd Switzerland, AlgoTherapeutix SAS France, Nanobiotix SA France, AmacaThera Inc Canada, Heat2Move, Resano GmbH, Esteve Pharmaceuticals SA. R. Baron has acted as a Speaker for Pfizer Pharma GmbH, Sanofi Aventis GmbH, Grünenthal GmbH, Mundipharma, Lilly GmbH, Desitin Arzneimittel GmbH, Teva GmbH, Bayer AG, MSD GmbH, Seqirus Australia Pty Ltd, Novartis Pharma GmbH, TAD Pharma GmbH, Grünenthal SA Portugal, Grünenthal Pharma AG Schweiz, Grünenthal B.V. Niederlande, Evapharma, Takeda Pharmaceuticals International AG Schweiz, Ology Medical Education Netherlands, Ever Pharma GmbH, Amicus Therapeutics GmbH, Novo Nordisk Pharma GmbH, Chiesi GmbH, Stada Mena DWC LLC Dubai, Hexal AG, Viatris, AstraZeneca GmbH, Sandoz.

## Appendix A. Supplemental digital content

Supplemental digital content associated with this article can be found online at http://links.lww.com/PAIN/C169 and http://links.lww.com/PAIN/C170.

## Supplementary Material

SUPPLEMENTARY MATERIAL
