# Supplementary material for: Genetic associations of neuropathic pain and sensory profile in a deeply phenotyped neuropathy cohort
Source: Pain. 2024 Oct 29;166(6):1354–68. doi: 10.1097/j.pain.0000000000003463 (PMC12067614; doi:10.1097/j.pain.0000000000003463)
Supplement: SUPPLEMENTARY MATERIAL [file jop-166-1354-s001.pdf]

Supplementary methods

Pain intensity measure harmonisation and imputation of missing pain scores (Supplementary data)

If the BPI average pain intensity score was not available, for those participants with neuropathic pain, a pain score was imputed from other measures. These other pain measures included:

- A bespoke question rating average pain intensity over 7 days
- Chronic Pain grade (CPG)[28]– Average pain intensity over 3 months
- PainDETECT[14] – Average pain intensity over 4 weeks
- Pain diary completed over 24 hours or 7 days.

A strong and significant correlations was observed between BPI average pain intensity and 7 Day average pain intensity (Rho=0.83), 7 Day pain diary (Rho=0.69), 24 Hour pain diary (Rho=0.56), Pain detect 4 week average (Rho=0.57), Chronic Pain Grade average 3 months (Rho=0.71). There was also a moderate correlation with DN4 (Rho=0.27) and a weak with TCSS (Rho=0.1). We removed instances that no other pain scores were available (Propane datasets) and from the other centres instances where only TCSS, DN4[3] or painDETECT were available.

A missing value analysis showed that were no significant dependencies between participants with present BPI average versus those with missing values, with the exception of a dependency on BMI. By inspecting all pairwise combinations between responders and non-responders, we consider the mechanism of missing values to be missing at random (MAR). Thus we proceeded to imputed the BPI average using Multiple Imputations by Chained Equations (MICE) and the predictive mean matching algorithm, considering as predictors the 24-hour and 7-day average pain diary, the 3-month average CPG, the 4-week average Pain Detect, the 7 Day average pain intensity, DN4 and TCSS. We used 100 rounds of multiple imputations after which we inspected the within imputations variance and ensured that between imputations variance was always smaller than the within imputations variance. The final imputed value was calculated as the median of the imputed values across the 100 rounds of multiple imputations. The pain score was recorded as missing if no score, BPI or otherwise, was recorded. Those participants were not excluded from the binary analysis (see below) as long as there was a clear record that they suffered from probable or definite neuropathic pain according to the NeupSIG criteria.

Table 1 and 2 listed all questionnaires and other specialised tests used in each cohort.

Table 1 Questionnaires of the DOLORisk protocol

Category	Questionnaire	Reference
**Demographics**	Age, gender, years in education, working status, weight, height	
**Characterisation of pain**	Presence and duration of pain	
**Family history**	Family history of chronic pain	
**Pain medication**	Currently taking pain medication	
	Brief Pain Inventory – Usefulness of medication	[9]
	Adherence to medication	
**Pain severity**	Chronic Pain Grade	[28]
	Brief Pain Inventory – Pain Severity	[9]
**Pain quality**	DN4 Questionnaire	[3]
	DN4 Examination	
	Neuropathic Pain Symptom Inventory	[4]
	PainDETECT	[14]
**Pain location**	List of locations	
	Body map	
**Pain interference**	PROMIS Pain Interference	[12]
**Pain catastrophizing**	Pain Catastrophizing Scale	[6]
**Health status and quality of life**	EQ-5D-5L	[25]
	PROMIS Depression 8a	[4]
	PROMIS Anxiety 8a	
	PROMIS Sleep Disturbance 8a	
	PROMIS Fatigue	

	Trauma	
**Disease specific (diabetic neuropathy)**	Michigan Neuropathy Screening Instrument	[18]
**Personality**	Ten Item Personality Inventory	[16]
	International Personality Item Pool (Emotional Stability)	[15]
**Lifestyle**	Smoking	[5]
	Alcohol	
	International Physical Activity Questionnaire	[10]

Table 2 Summary of tests performed

Cohort	Neurological examination	TCSS	TNSn	Skin biopsy	QST	NCS	EEG	Threshold tracking	CPM
Diabetes	X	X		X	X	X	X	X	X
Traumatic nerve injury	X	X				X		X	
Surgery	X				X			X	X
Chemotherapy	X		X		X	X		X	
Extreme phenotypes	X	X			X	X		X	

TCSS- Toronto clinical scoring system; TNSn- Total Neuropathy Score – Nurse; QST- Quantitative sensory testing; EEG - Electroencephalography; CPM- Conditioned pain modulation

A number of additional cohorts

A number of additional cohorts in which neuropathic pain phenotype could be harmonised were included alongside the DOLORisk cohorts. If the BPI score was not available as a measure of pain intensity, for those participants with neuropathic pain, a pain score was imputed from other measures which included a bespoke question rating average pain intensity over 7 days, Chronic Pain grade (CPG) – Average pain intensity over 3 months, painDETECT – Average pain intensity over 4 weeks or pain diary completed over 24hours or 7 days. The pain score was recorded as missing if no score, BPI or otherwise, was recorded. Those participants were not excluded as they were included in the binary analysis of neuropathic pain versus control.

International Diabetes Neuropathy Consortium (IDNC)

International Diabetes Neuropathy Consortium (IDNC) is a multi-centre cross-sectional study designed to understand polyneuropathy in type 2 diabetes mellitus. Participants were recruited from the Danish Center for Strategic Research in type 2 diabetes cohort (DD2). Included in the DD2 cohort are more than 9,000 patients with type 2 diabetes[8,17]. A detailed description of the cohort and the primary clinical results are given elsewhere[24].

A subgroup of the cohort was sent a questionnaire, and invitations were sent to the responders of this questionnaire. Study participants with diabetes mellitus were recruited in Aarhus and

Odense, Denmark. Central Denmark Region Committees on Health Research Ethics (1-10-72-130-16) approved the study. The ClinicalTrials.gov identifier is NCT02947828.

The IDNC invited a subsample of the DD2 cohort to attend clinic for a detailed physical examination. Participants were assessed by trained neurologists for neuropathy[11,13] and neuropathic pain[13], using the same grading systems as were used in the DOLORisk extended cohort (these studies were co-designed at inception). Two measures of pain intensity were available, asking the participants to assess their pain in the feet on average respectively over the past 24 hours and the past 7 days, using a visual analogue scale (VAS). As in DOLORisk, we excluded participants with possible neuropathic pain, and those with no pain and possible neuropathy. Furthermore, we excluded participants in which there was a conflict between case definition and pain scores: participants coded with probable or definite neuropathic pain, but a pain intensity score in the feet of 0 both over the past 24 hours and the past 7 days or participants with a painless neuropathy, a DN4 score superior or equal to 3, and a pain intensity score in the feet superior to 0 over 24 hours and 7 days.

Exploring the genetics of neuropathic pain study (GeNeup)

The GeNeup study is a multi-centre cross-sectional study designed to understand the genetics of neuropathic pain. Participants are recruited from centres across Norway. Patients referred for suspected distal symmetric polyneuropathy are included if they are between 18 and 70 years of age, able to consent, not too unwell to participate and do not have an acute inflammatory polyneuropathy. Ethical approval was granted from Regional Committee for Medical Research Ethics, ref no 2017/1593/REK sor-ost C. The ClinicalTrials.gov identifier is NCT03862365.

The protocol was broadly aligned with the DOLORisk extended protocol, as far as Norwegian translations of the questionnaires could be obtained. The assessments of neuropathy and neuropathic pain were fully aligned with the DOLORisk method. Pain intensity was measured using the short form of the Brief Pain Inventory (BPI)[28] Average pain intensity item.

Probing the Role of Sodium Channels in Painful Neuropathies (PROPANE)

The PROPANE study aimed to identify new variants in genes encoding for the sodium channel subunits expressed in the peripheral nociceptive pathway, to provide diagnostic evidence in patients affected by idiopathic painful small fibre neuropathy, and to explain the occurrence of neuropathic pain in patients affected by diabetic neuropathy. Details of ethics permissions are available in Almomani et al[1].

To be eligible to the study, patients had to meet the following inclusion criteria: (1) A diagnosis of sensory neuropathy, based on clinical and neurological examination, nerve conduction studies (NCS), quantitative sensory testing (QST) and/or skin biopsy findings[5] with diabetic or idiopathic aetiology; (2) Definite or probable NeuP based on the definition and grading system of Treede et al.[26] for more than one year or no pain;(3) Age of 18 years or older. Participants with possible NeuP were excluded.

The diagnosis of DPN was established based on clinically confirmed diabetes, clinical signs of sensory neuropathy, and abnormal NCS. If NCS was normal, decreased IENFD was required. SFN patients were included if the diagnosis of SFN was established based on clinical signs of sensory neuropathy, decreased IENFD on skin biopsy and/or abnormal QST, with no evidence of large nerve fibre damage on neurological examination and NCS. If IENFD was normal, abnormal QST was required to confirm SFN[1].

The painful neuropathy group consisted of DPN or SFN patients with NeuP for >1 year and a mean pain intensity ≥ 4 on the pain intensity numerical rating scale (PI-NRS) at the screening visit. The painless neuropathy group consisted of DPN or SFN patients with no pain (PI-NRS of 0) or NeuP for >1 year and a mean pain intensity < 4 without analgesic drugs on PI-NRS[1].

DNA of participants from three sub-groups were shared with DOLORisk:

- Participants with diabetic polyneuropathy (DPN) recruited in Germany/Maastricht: The PROPANE protocol used measures of pain intensity during the day and at night. To harmonise with the DOLORisk data (in which no differentiation was made between pain in the day versus night), average pain intensity was re-calculated as the average of "Mean pain during the day" and "Mean pain at night". The majority of painless participants uniformly reported pain intensity scores of 0 and so could be matched to the DOLORisk control group. We excluded those in which group allocation conflicted with pain scores: painless participants with non-zero mean pain intensity scores from the binary analysis; Painful participants with mean pain intensity of 0 were excluded from the binary analysis.
- Participants with diabetic polyneuropathy recruited in Milan: These samples were recruited at Institute of Cardiovascular Sciences, Cardiac Centre, Faculty of Medical and Human Sciences, The University of Manchester, United Kingdom. For this group, we did not have access to the individual pain intensity scores. The case definition included having a pain intensity score of 4 and above and so could be included in neuropathic pain cases.

- Participants with small fibre neuropathy recruited in Milan: The pain score available to us in this group was a numerical rating scale from a pain diary over 24 hours. We classified participants into the painless group if they reported a pain intensity of 0 over 24 hours, and into the painful group otherwise. For participants with multiple visits, we only considered the pain intensity reported on the first visit, as this was when the diagnosis was first made. We were not able to impute an average pain intensity score from the 24-hour NRS, so the participants were not included in the quantitative pain intensity analysis.

Clinical Phenotyping and Genotyping of HIV-Associated Sensory Neuropathy: The HIV-POGO Study (HIV-POGO)

HIV-POGO recruited a cohort of HIV patients with and without HIV-SN in order to understand risk factors for the development of HIV-SN and neuropathic pain. Patients for the HIV cohort were recruited from HIV outpatient clinics associated with Chelsea & Westminster NHS Foundation Trust and by advertisement to national HIV charities. Subjects were required to be aged 18 or older and have a serological diagnosis of HIV. In addition to a DN4i score ≥3/7, only subjects displaying one sign and one symptom on the Clinical HIV-associated neuropathy tool (CHANT), as a case definition of neuropathy[29] were included. The study was approved by England's National Research Ethics Service (14/LO/1574). The BPI average was available as a measure of pain intensity, aligned with the DOLORisk cohort.

OPTION - DM

OPTION-DM was a multicentre, randomised, double-blind, centre-stratified, multi-period crossover trial with active washout in patients with DPNP from primary and secondary care at 13 UK centres. The Yorkshire and the Humber Sheffield Research Ethics Committee (16/YH/0459) approved the trial. The full protocol is described in Selvarajah et al.,[23], and was partially aligned with the DOLORisk protocol. The inclusion criteria included:

- Bilateral distal symmetrical diabetic polyneuropathy confirmed by the modified Toronto Clinical Neuropathy Score (mTCNS) score > 5 at screening visit
- Bilateral distal symmetrical neuropathic pain confirmed by the Douleur Neuropathique 4 (DN4)[27] questionnaire score of ≥ 4 at screening visit
- Mean total pain intensity of at least 4 on an 11-point numeric rating scale (with 0 being 'no pain' and 10 'worst pain imaginable') during 1 week off pain medications (Baseline Period)

The screening visit also included a full physical and neurological assessment to ensure the presence of a distal symmetrical polyneuropathy that started in the feet. All OPTION participants for whom we received samples and phenotypic data (which we confirmed met criteria for probable neuropathic pain) were thus included in our case group for all genetic analyses (binary and pain intensity quantitative outcome).

Pain intensity measures were available at several time points:

- A 7-day pain diary before medication washout
- A 7-day pain diary at baseline
- The BPI Short Form pain severity score at week 0

We decided to take the average of the daily pain intensity scores over 7 days before medication washout, in order to ensure that we had a "naïve" pain intensity score – i.e. not influenced by the washout period or the treatment dispensed as part of the clinical trial. We considered this 7-day pain diary score to be equivalent to the DOLORisk bespoke item measuring average pain intensity over 7 days, and used this to perform the imputation of the pain intensity score.

GWAS Genotyping

Genotyping was performed at the Lund University Diabetes Centre (LUDC), Department of Clinical Sciences, Diabetes and Endocrinology (Lund, Sweden). A total of 2740 samples were genotyped using the ChipArray Infinium Global Screening Array-24 v.2.0/v3.0 assay Infinium HTS (1st batch, n=672 using v2.0 and 2nd batch, n=2068 using v.3.0) and run on Illumina Iscan system. Standard quality control steps (QC) as described by a previous study were applied[2]. Imputation was carried out using the Michigan Imputation Server, with the European population serving as the reference (panel HRC r1.1 2016). Only individuals of European ancestry and unrelated to the second degree and only common SNPs with a Minor Allele Frequency (MAF) greater than 0.01 and good imputation quality (R^2>0.4) were included in further analyses. A final dataset consisting of 7,837,857 SNPs and 2,467 individuals with available phenotypic information was obtained for subsequent analyses.

In order to investigate the association of rare genomic variants and neuropathic pain we carried out Whole Exome Sequencing for 1,702 DOLORisk participants. Exome sequencing was performed in the Wellcome Centre for Human Genetics using the Twist Human Core Exome EF Multiplex Complete Kit as the basis, but with additional spiked-in probes to maximise capture of the 45 DOLORisk target genes (Supplementary Table 3). Samples were sequenced

in two batches/tranches of 675 and 1,027 respectively, producing 1,715 sequencing runs with some duplicate runs to achieve the required sequencing depth (Table 3).

Table 3. Coverage of WES

	45 target genes	Exome wide
Tranche 1	84.8x	81.1x
Tranche 2	90.6x	86.1x

After joint genotype calling, data was left aligned, normalised, filtered and annotated with the dbsnp_138.b37 and gnomad.exomes.r2.1.1 datasets. Multiallelic sites were split into bi-allelic records, alleles not seen were removed, and SNPs and INDELS with at least one non-major allele were considered for downstream analysis.

To determine ethnicity, we have anchored and projected our data to the latest (phase 3) release of the 1000 genomes project. Then we considered the first 10 principal components of the merged data to predict ethnicity using linear discriminant analysis. The majority of our cohort clustered with the European population of the 1,000 genome project. We subsequently limited our analysis to the 1,531 individuals with European ancestry in our cohort, Table 4, Supplementary Figure 1. Joint genotype calling was done using GATK version 4.1.7.0 with standard options[27]. WES data was further pre-processed, left aligned, normalised, filtered and annotated using BCFtools 1.10.2. Sequencing data was checked against the reference genome human_g1k_v37. Multiallelic sites were split into bi-allelic records, alleles not seen were removed, and SNPs and INDELS with at least one non major allele were considered for downstream analysis. Variants were annotated with the dbsnp_138.b37 and gnomad.exomes.r2.1.1 datasets. Deviation from Hardy Weinberg Equilibriums were tested using the Fisher's Exact test. Data was filtered for sequencing depth (DP > 3) and genotyping quality (GQ > 20), variants with > 0.01 and individuals with > 0.02 missing calls were removed (n = 12). We further removed variants with an average sequencing depth < 10. Further QC was carried out using PLINK version 1.90b6.26[22]. Composite sample ids were created in the form "FID_IID", chromosome X was split using the b37 reference. We removed the duplicate runs with lower call rate (n = 12) and removed individuals with sex discrepancies based on chromosome X inbreeding (n = 22). With a subset of autosomal SNPs, with MAF>=0.05, HWE p > 1e-5 and in linkage disequilibrium (--indep-pairwise 50 5 0.2) we checked for heterozygocity rates and removed outlying individuals (n = 28), checked for cryptic relatedness

and from each pair with a pi^>0.2 removed the one with the lowest call rate (n = 12) and calculated genetic principal components.

Table 4. Predicted ethnicity based on projections of the WES data on the 1000 genomes project.

Europeans	Africans	Asians	Indian Telugu in the UK	Sri Lankan Tamil in the UK
1,531	25	50	11	12

GWAS analyses

After QC and pre-process we tested for associations with painful versus painless neuropathy. In each case we used sex, age, PC 1-4 and batch as covariates (model 1) and when looking at diabetic participants only we also considered sex, age, PC 1-10, Toronto Clinical Scoring System (TCSS) and batch as covariates (model 2). After removing samples with missing covariates, we considered 1458 participants (1,026 cases and 432 controls), out of which 1,048 were diabetics. Phenotypes were transformed with the inverse-normal transformation. Table 5 summarised the QC details.

Table 5. Number of participants excluded during QC steps

	Whole Exome Sequencing	Genotyping Arrays
Total genotyped	1,715	2,740
QC steps	Individuals removed	
Missingness > 0.02	12	95
Duplicates (runs with lower call rate were removed)	12	32
Sex discrepancies	22	92
Heterozygocity outliers	28	26
Cryptic relatedness	12	45
Non-European	98	66
Passed QC		
Total	1,531	2,417
Removed due to missing covariates M1	73	26
Removed due to missing covariates M2	289	1,087
Considered in analysis M1	1,458	2,391
Considered in analysis M2	1,242	1,330

Whole Exome Sequencing

Group-wise association were tested for rare variants on a subset of the 45 DOLORisk genes that carried rare variants. 10/45 genes had rare variants survived filtering: *SPTLC1*, *PIEZO2*, *NTRK1*, *MMP1*, *TRPM8*, *HCN3*, *OPRM1*, *SCN3A*, *SCN9A*, *SCN10A*. We tested for gene-wise associations using a variant component optimal test (SKAT-O) and reverse regression with a Wilcoxon test[21]. For these analyses we only considered un-related individuals (1,429 individuals, 145 diabetics). We also used a subset of high-quality variants in linkage disequilibrium to calculate a kinship matrix that we used in the Efficient Mixed-Model Association eXpedited (EMMAX) burden test[19]. For these analyses we considered all participants. In all cases we removed participants with congenital insensitivity to pain from controls and we filtered for variants that were either pathogenic or likely pathogenic on ClinVar[20] or being known in dbSNP, protein coding, with GNOMAD[7] AF in NFE < 0.01 classified as damaging or deleterious or Hich Confidence loss of function or other splice by two of PolyPhen, SIFT and Loftee and not found to be tolerate or benign by any tool and not having low impact according to VEP. We further considered two minor allele counts thresholds, min MAC =1 and min MAC = 3.

Ethical approvals

All participants provided written informed consent in accordance with the Declaration of Helsinki. Details of the ethical approvals are given here.

"DOLORisk: understanding risk factors and determinants for neuropathic pain", Imperial College London has been approved by London—Bromley Research Ethics Committee (REC: 16/LO/1470, IRAS project ID: 209707). "Pain in Neuropathy Study" amended for DOLORisk University of Oxford UK, has been approved by the National Research Ethics Service—West London REC 3 (No.: 10/H07056/35). "Understanding Risk Factors and Determinants for Neuropathic Pain", Technion—Israel Institute of Technology, has been approved by the Helsinki Committee of Rambam Health Care Campus (Reference: 0052–15-RNB, NCT: NCT02402361). "Facteurs prédictifs génétiques, neurophysiologiques et psychologiques de la douleur chronique neuropathique après chirurgie pour cancer du sein". INSERM, CPP 8 Ile de France. N'ID RCP N°ID RCB 2016-A00225-46. The International Diabetic Neuropathy Consortium study was approved by the Central Denmark Region Committees on Health Research Ethics (1-10-72-130-16). The prospective breast and thoracic surgery and chemotherapy DOLORisk studies in Aarhus were approved by the Central Denmark Region

Committees on Health Research Ethics (1-10-72-23-17; 1-10-72-254-16; 1-10-72-359-15). N° ID RCB: 2016-A00225-46 (CPP IDF 8). “DOLORisk: understanding risk factors and determinants for neuropathic pain the influence of sensory phenotype on the risk of developing neuropathic pain”, D454/16 approved 20th June, Christian Albrechts University Kiel. The trial “Comparison of amitriptyline supplemented with pregabalin, pregabalin supplemented with amitriptyline, and duloxetine supplemented with pregabalin for the treatment of diabetic peripheral neuropathic pain (OPTION-DM)” was approved by the Yorkshire and the Humber Sheffield Research Ethics Committee (16/YH/0459). “Clinical Phenotyping and Genotyping of HIV-Associated Sensory Neuropathy: The HIV-POGO Study (HIV-POGO)” was approved by England’s National Research Ethics Service (14/LO/1574). “Probing the role of sodium channels in painful neuropathies (PROPANE)” received ethical approval, Institute Carlo Besta, 11th Dec 2013. The Norwegian “Exploring the genetics of neuropathic pain” study was approved by Regional Ethical Committee (2017#22544).

References

[1]. Almomani R, Sopacua M, Marchi M, Ślęczkowska M, Lindsey P, de Greef BT, Hoeijmakers JG, Salvi E, Merkies IS, Ferdousi M. Genetic profiling of sodium channels in diabetic painful and painless and idiopathic painful and painless neuropathies. International journal of molecular sciences 2023;24(9):8278.

[2]. Anderson CA, Pettersson FH, Clarke GM, Cardon LR, Morris AP, Zondervan KT. Data quality control in genetic case-control association studies. Nature protocols 2010;5(9):1564-1573.

[3]. Bouhassira D, Attal N, Alchaar H, Boureau F, Brochet B, Bruxelle J, Cunin G, Fermanian J, Ginies P, Grun-Overdyking A. Comparison of pain syndromes associated with nervous or somatic lesions and development of a new neuropathic pain diagnostic questionnaire (DN4). pain 2005;114(1-2):29-36.

[4]. Bouhassira D, Attal N, Fermanian J, Alchaar H, Gautron M, Masquelier E, Rostaing S, Lanteri-Minet M, Collin E, Grisart J. Development and validation of the neuropathic pain symptom inventory. Pain 2004;108(3):248-257.

[5]. Campbell A, Kerr S, Porteous D. Generation Scotland SFHS Data Dictionary, 2006-2011. University of Edinburgh School of Molecular, Genetic and Population Health Sciences Institute of Genetics and Molecular Medicine 2018;10.

[6]. Cella D, Riley W, Stone A, Rothrock N, Reeve B, Yount S, Amtmann D, Bode R, Buysse D, Choi S. The Patient-Reported Outcomes Measurement Information System (PROMIS) developed and tested its first wave of adult self-reported health outcome item banks: 2005–2008. Journal of clinical epidemiology 2010;63(11):1179-1194.

[7]. Chen S, Francioli LC, Goodrich JK, Collins RL, Kanai M, Wang Q, Alföldi J, Watts NA, Vittal C, Gauthier LD. A genome-wide mutational constraint map quantified from variation in 76,156 human genomes. BioRxiv 2022:2022.2003. 2020.485034.

[8]. Christensen DH, Nicolaisen SK, Berencsi K, Beck-Nielsen H, Rungby J, Friborg S, Brandslund I, Christiansen JS, Vaag A, Sørensen HT. Danish Centre for Strategic Research in Type 2 Diabetes (DD2) project cohort of newly diagnosed patients with type 2 diabetes: a cohort profile. BMJ open 2018;8(4):e017273.

[9]. Cleeland C, Ryan K. Pain assessment: global use of the Brief Pain Inventory. Annals of the Academy of Medicine, Singapore 1994;23(2):129-138.

[10]. Craig CL, Marshall AL, Sjöström M, Bauman AE, Booth ML, Ainsworth BE, Pratt M, Ekelund U, Yngve A, Sallis JF. International physical activity questionnaire: 12-country reliability and validity. Medicine & science in sports & exercise 2003;35(8):1381-1395.

[11]. Dyck PJ, Albers JW, Andersen H, Arezzo JC, Biessels GJ, Bril V, Feldman EL, Litchy WJ, O'Brien PC, Russell JW. Diabetic polyneuropathies: update on research definition, diagnostic criteria and estimation of severity. Diabetes/metabolism research and reviews 2011;27(7):620-628.

[12]. Feldman EL, Stevens M, Thomas P, Brown M, Canal N, Greene D. A practical two-step quantitative clinical and electrophysiological assessment for the diagnosis and staging of diabetic neuropathy. Diabetes care 1994;17(11):1281-1289.

[13]. Finnerup NB, Haroutounian S, Kamerman P, Baron R, Bennett DL, Bouhassira D, Cruccu G, Freeman R, Hansson P, Nurmikko T. Neuropathic pain: an updated grading system for research and clinical practice. Pain 2016;157(8):1599-1606.

[14]. Freynhagen R, Baron R, Gockel U, Tölle TR. Pain DETECT: a new screening questionnaire to identify neuropathic components in patients with back pain. Current medical research and opinion 2006;22(10):1911-1920.

[15]. Goldberg LR. A broad-bandwidth, public domain, personality inventory measuring the lower-level facets of several five-factor models. Personality psychology in Europe 1999;7(1):7-28.

[16]. Gosling SD, Rentfrow PJ, Swann Jr WB. A very brief measure of the Big-Five personality domains. Journal of Research in personality 2003;37(6):504-528.

[17]. Gylfadottir SS, Itani M, Krøigård T, Kristensen AG, Christensen DH, Nicolaisen SK, Karlsson P, Callaghan BC, Bennett DL, Andersen H. Diagnosis and prevalence of diabetic polyneuropathy: a cross-sectional study of Danish patients with type 2 diabetes. European journal of neurology 2020;27(12):2575-2585.

[18]. Herdman M, Gudex C, Lloyd A, Janssen M, Kind P, Parkin D, Bonsel G, Badia X. Development and preliminary testing of the new five-level version of EQ-5D (EQ-5D-5L). Quality of life research 2011;20:1727-1736.

[19]. Kang HM, Sul JH, Service SK, Zaitlen NA, Kong S-y, Freimer NB, Sabatti C, Eskin E. Variance component model to account for sample structure in genome-wide association studies. Nature genetics 2010;42(4):348-354.

[20]. Landrum MJ, Lee JM, Benson M, Brown GR, Chao C, Chitipiralla S, Gu B, Hart J, Hoffman D, Jang W. ClinVar: improving access to variant interpretations and supporting evidence. Nucleic acids research 2018;46(D1):D1062-D1067.

[21]. Lee S, Emond MJ, Bamshad MJ, Barnes KC, Rieder MJ, Nickerson DA, Christiani DC, Wurfel MM, Lin X. Optimal unified approach for rare-variant association testing with application to small-sample case-control whole-exome sequencing studies. The American Journal of Human Genetics 2012;91(2):224-237.

[22]. Purcell S, Neale B, Todd-Brown K, Thomas L, Ferreira MA, Bender D, Maller J, Sklar P, De Bakker PI, Daly MJ. PLINK: a tool set for whole-genome association and population-based linkage analyses. The American journal of human genetics 2007;81(3):559-575.

[23]. Selvarajah D, Petrie J, White D, Julious S, Bortolami O, Cooper C, Bradburn M, Loban A, Bowler H, Swaby L. Multicentre, double-blind, crossover trial to identify the Optimal Pathway for TreatIng neurOpathic paiN in Diabetes Mellitus (OPTION-DM): study protocol for a randomised controlled trial. Trials 2018;19:1-12.

[24]. Sørensen HT, Friborg S, Rungby J, Christensen JS, Vaag A, Beck-Nielsen H. The Danish national type 2 diabetes cohort–the DD2 study, Vol. 4: Taylor & Francis, 2012. pp. 1-5.

[25]. Sullivan MJ, Bishop SR, Pivik J. The pain catastrophizing scale: development and validation. Psychological assessment 1995;7(4):524.

[26]. Treede R-D, Jensen TS, Campbell J, Cruccu G, Dostrovsky J, Griffin J, Hansson P, Hughes R, Nurmikko T, Serra J. Neuropathic pain: redefinition and a grading system for clinical and research purposes. Neurology 2008;70(18):1630-1635.

[27]. Van der Auwera GA, O'Connor BD. Genomics in the cloud: using Docker, GATK, and WDL in Terra: O'Reilly Media, 2020.

[28]. Von Korff M, Ormel J, Keefe FJ, Dworkin SF. Grading the severity of chronic pain. Pain 1992;50(2):133-149.

[29]. Woldeamanuel YW, Kamerman PR, Veliotes DG, Phillips TJ, Asboe D, Boffito M, Rice AS. Development, validation, and field-testing of an instrument for clinical assessment of HIV-associated neuropathy and neuropathic pain in resource-restricted and large population study settings. PLoS One 2016;11(10):e0164994.

Supplementary Figure 1. Projection of WES data on the 1000 genomes project. Ethnicity is genetically determined and colour coded

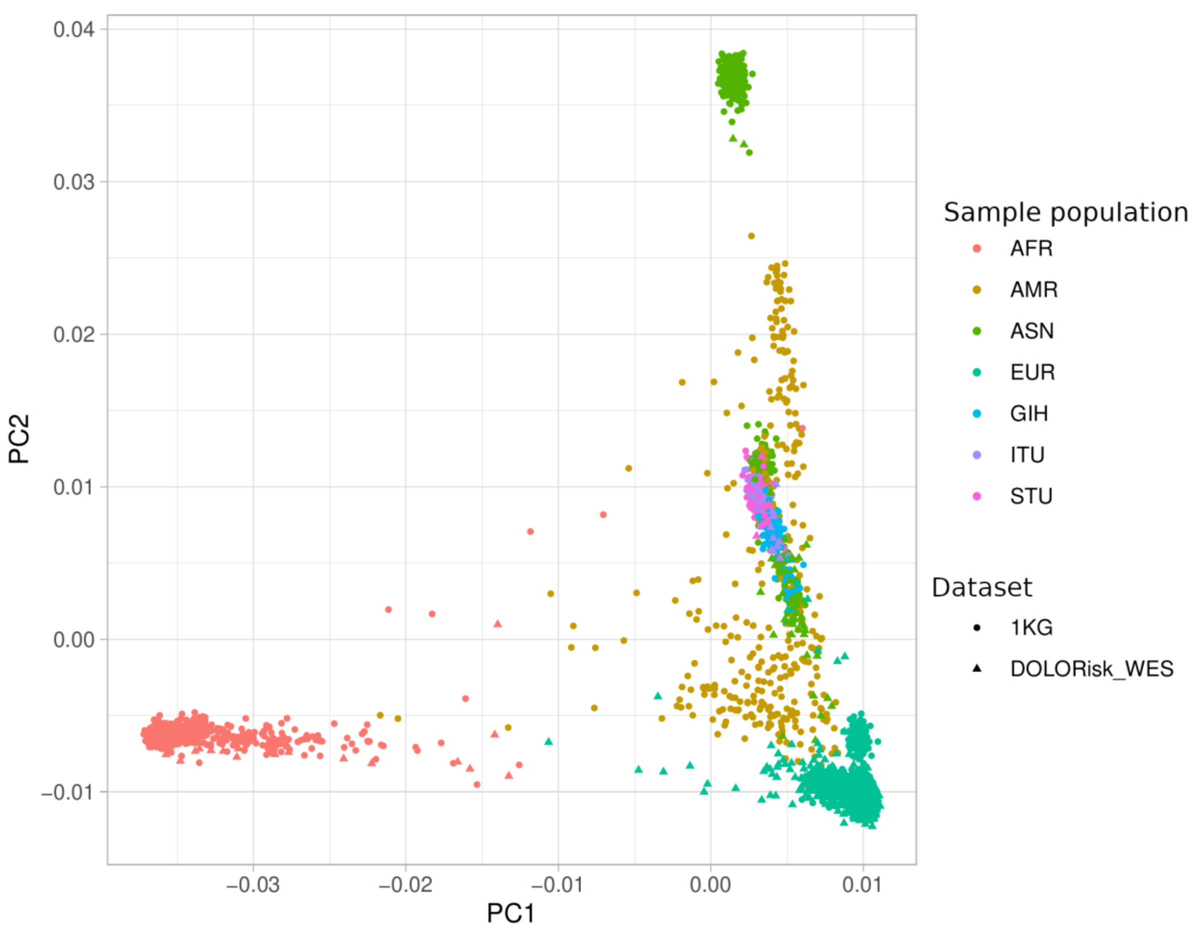

Supplementary figure 2. GWAS for binary measures of neuropathic pain vs no pain in whole cohort.

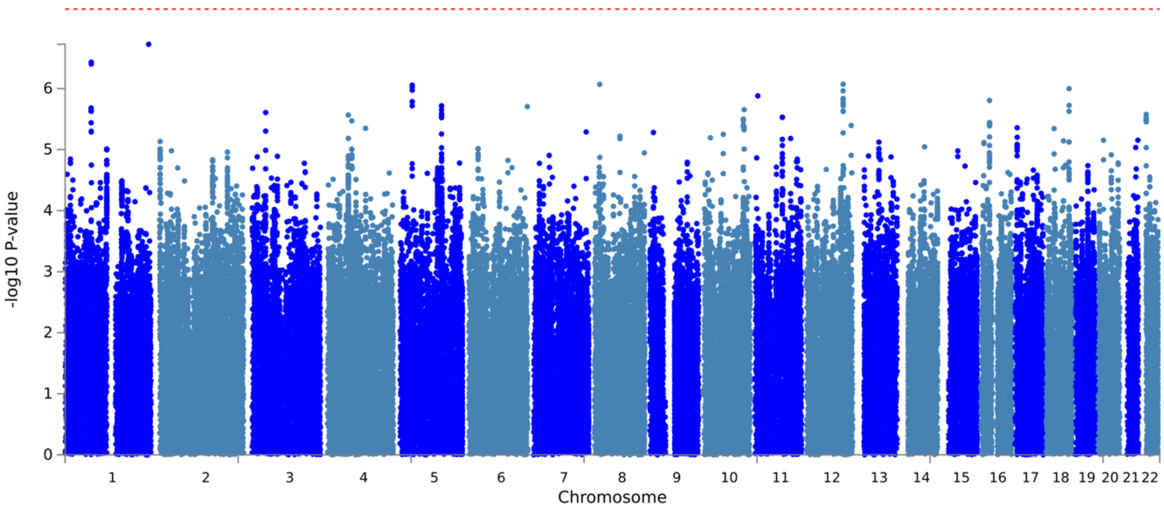

GWAS for binary measures of neuropathic pain vs no pain in whole cohort.

Shown is Manhattan plot at the SNP-level, genome-wide significant level is highlighted by a horizontal red line at a threshold of 5×10^{-8}. See suggestive results in Supplementary Table 12.

Supplementary figure 3. GWAS for quantitative measures of neuropathic pain vs no pain in whole cohort

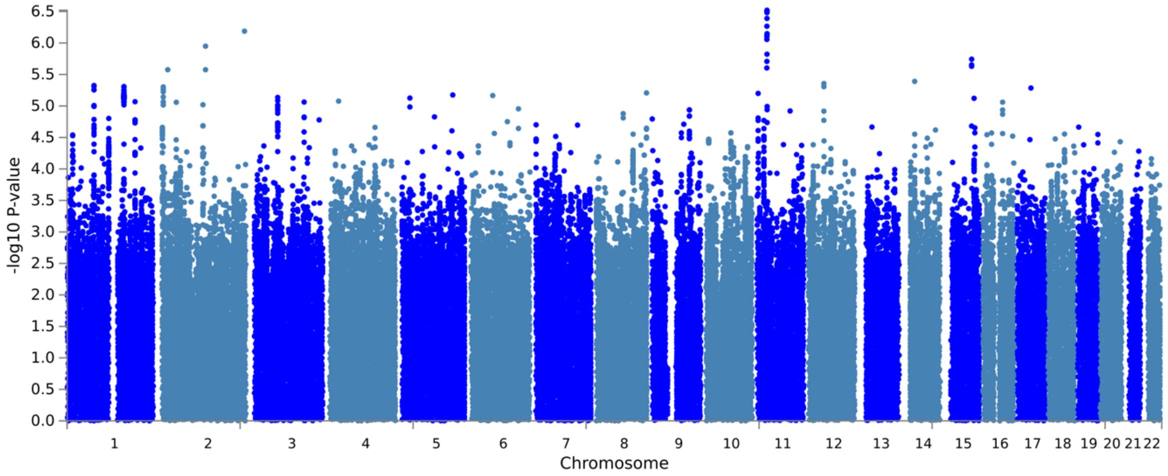

GWAS for neuropathic pain intensity for the whole cohort. Shown is Manhattan plot at the SNP-level. See suggestive results in Supplementary Table 12.

Supplementary figure 4. GWAS for binary measures of neuropathic pain vs no pain in diabetic cohort.

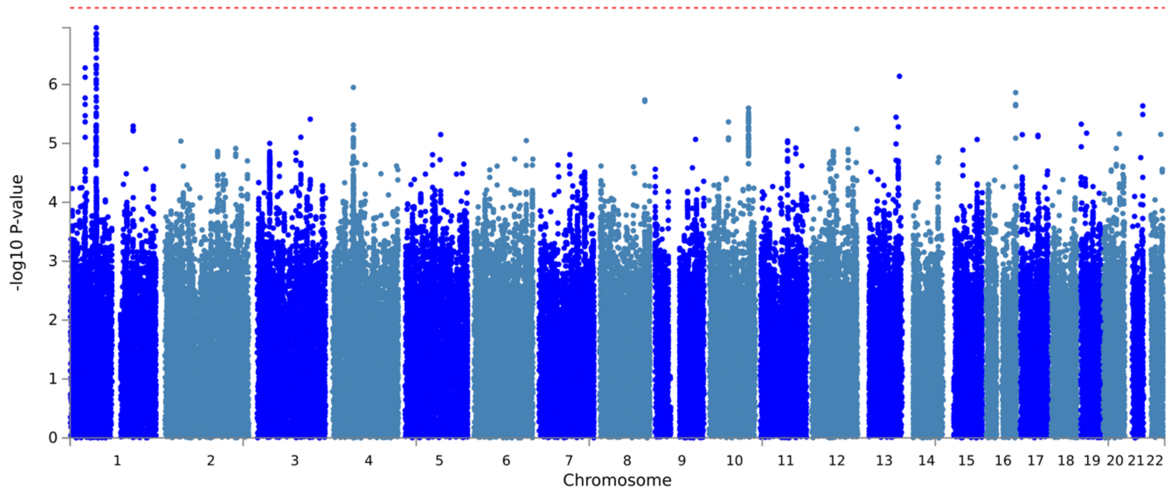

GWAS for binary measures of neuropathic pain vs no pain in diabetic cohort. Shown is Manhattan plot at the SNP-level, genome-wide significant level is highlighted by a horizontal red line at a threshold of 5×10^{-8}. See suggestive results in Supplementary Table 12.

Supplementary figure 5. GWAS for binary measures of neuropathic pain vs no pain in diabetic cohort with additional TCSS as covariates

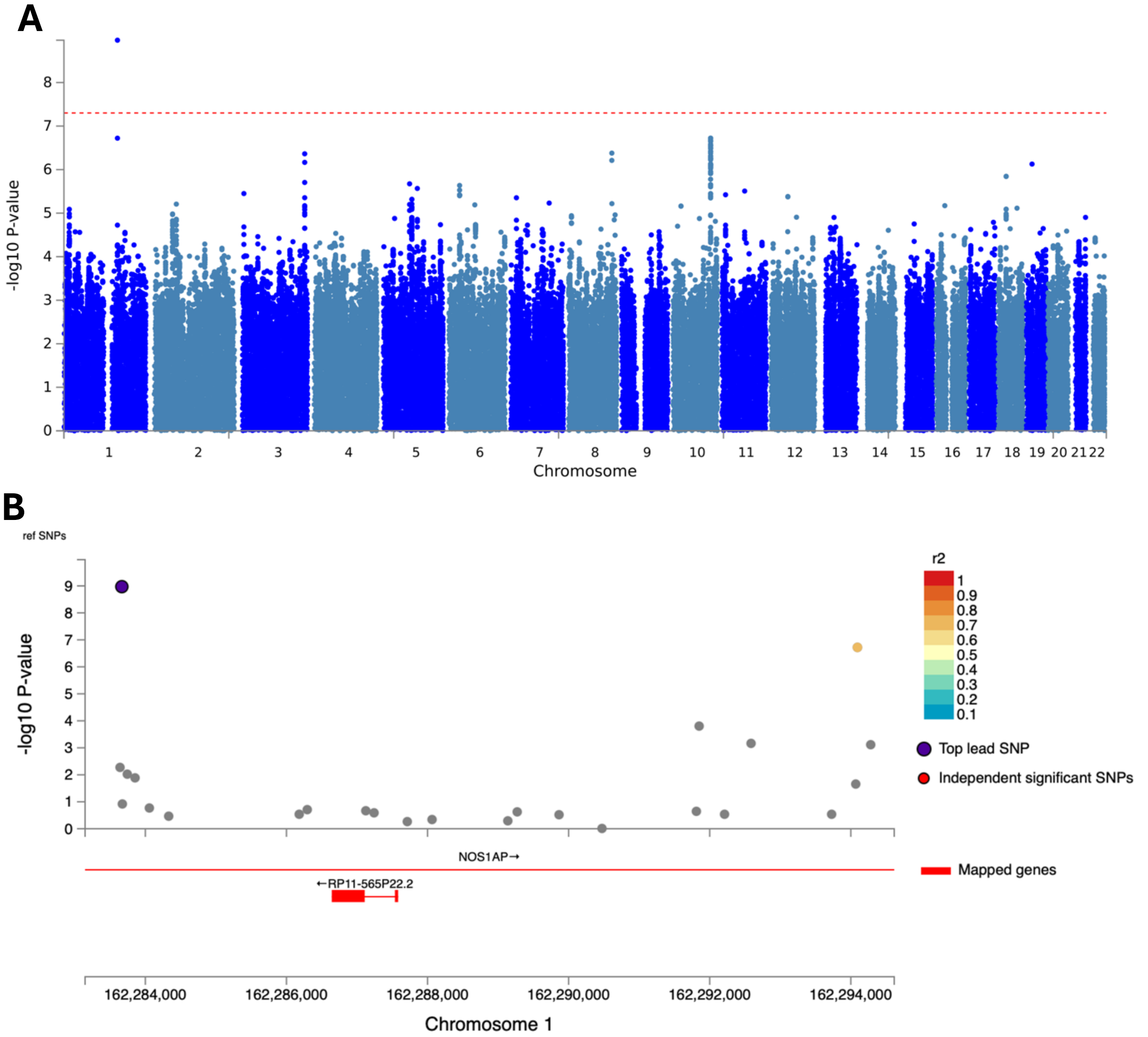

GWAS for binary measures of neuropathic pain vs no pain in diabetic cohort with additional TCSS as covariate. (A) Shown is Manhattan plot at the SNP-level, genome-wide significant level is highlighted by a horizontal red line at a threshold of 5 x 10^{-8}. **(B)** Regional plot for the top lead SNP in the GWAS. Each SNP is colour-coded based on the highest r^2 to the top independent significant SNP.

Supplementary figure 6. GWAS for quantitative measures of neuropathic pain vs no pain in diabetic cohort with additional TCSS as covariates

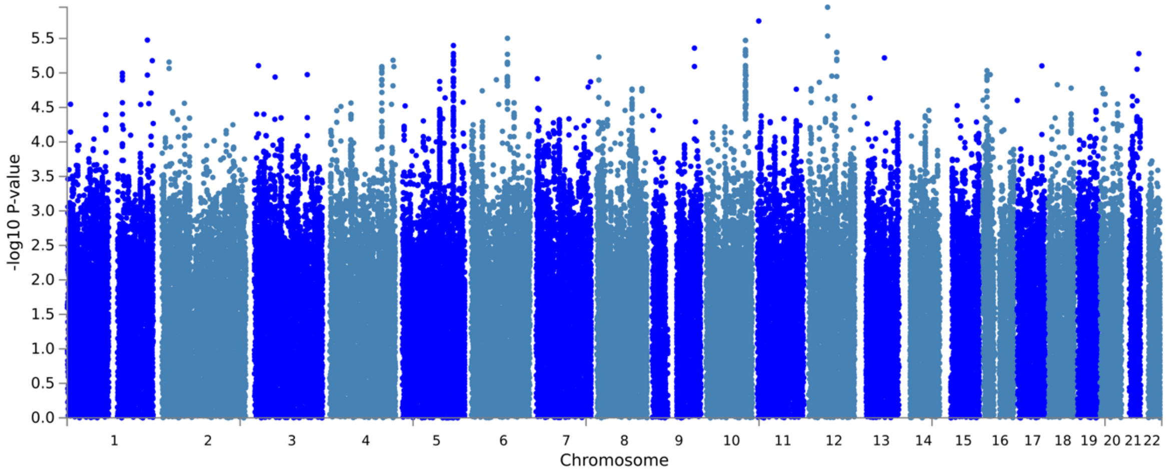

GWAS for quantitative measures of neuropathic pain vs no pain in diabetic cohort with additional TCSS as covariates. Shown is Manhattan plot at the SNP-level. See suggestive results in Supplementary Table 12.

Supplementary figure 7. Manhattan plot (gene-based test) for binary measures of neuropathic pain vs no pain in diabetic cohort (adjusting for TCSS) revealed one significant gene

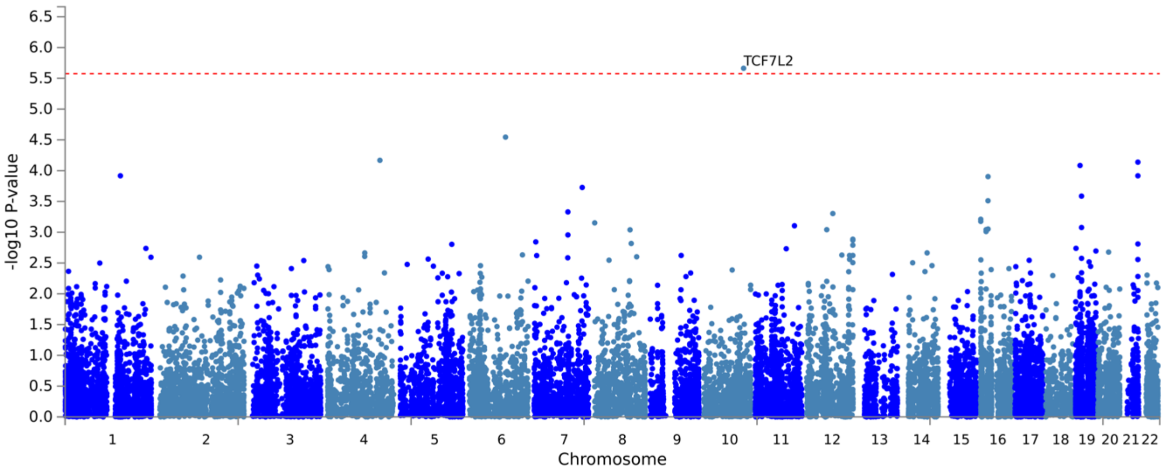

Manhattan plot (gene-based test) for binary measures of neuropathic pain vs no pain in diabetic cohort (adjusting for TCSS) revealed one significant gene. Input SNPs are mapped to 18829 protein coding genes. Genome wide significance (red dashed line in the plot) is defined at P = 0.05/18829 = 2.655×10^{-6}.

Supplementary figure 8. KCNT2 *and* CFH *Circos plots of chromatin interactions and eQTLs.*

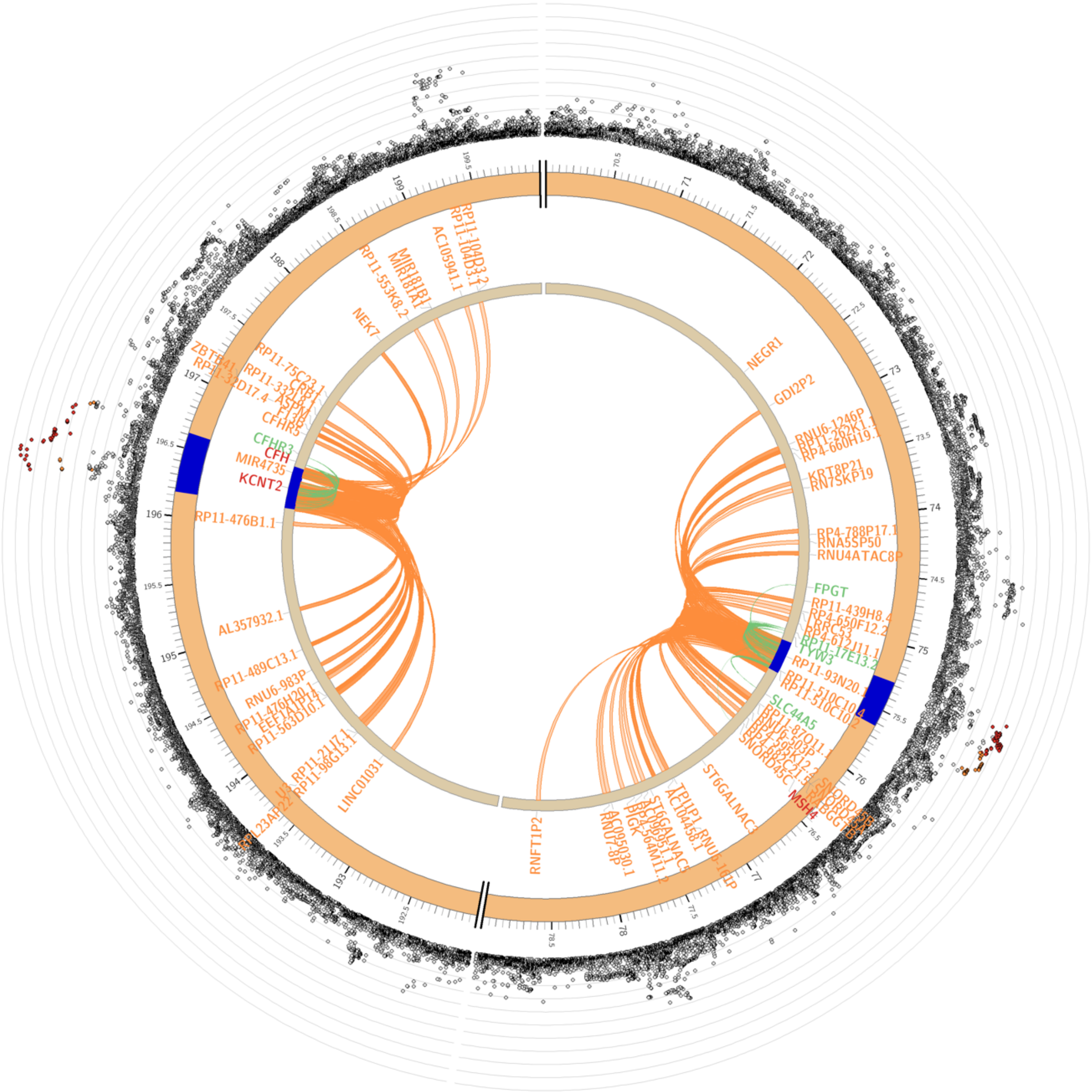

Circos plots of chromatin interactions and eQTLs. *Manhattan plot: The most outer layer. Only SNPs with P < 0.05 are displayed. Chromosome ring: The second layer. Genomic risk loci are highlighted in blue.Mapped genes by chromatin interactions or eQTLs: If the gene is mapped only by chromatin interactions or only by eQTLs, it is colored orange or green, respectively. When the gene is mapped by both, it is colored red. Chromatin interaction links: Links colored orange are chromatin interactions. eQTL lilnks: Links colored green are eQTLs.*

Supplementary figure 9. NOS1AP *Circos plots of chromatin interactions and eQTLs*

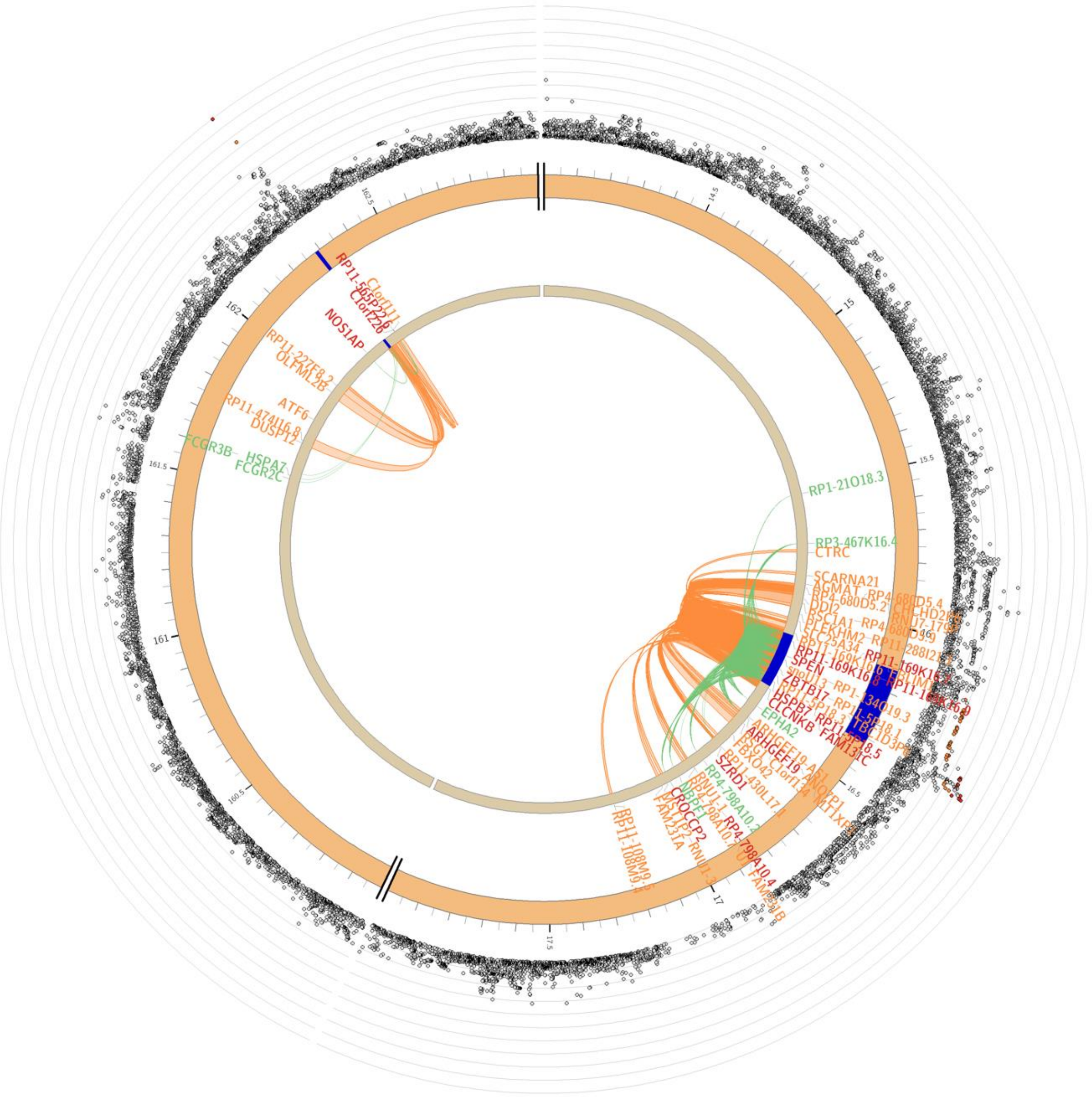

Circos plots of chromatin interactions and eQTLs. *Manhattan plot: The most outer layer. Only SNPs with P < 0.05 are displayed. Chromosome ring: The second layer. Genomic risk loci are highlighted in blue. Mapped genes by chromatin interactions or eQTLs: If the gene is mapped only by chromatin interactions or only by eQTLs, it is coloured orange or green, respectively. When the gene is mapped by both, it is coloured red. Chromatin interaction links: Links coloured orange are chromatin interactions. eQTL lilnks: Links coloured green are eQTLs.*

Supplementary figure 10. Irritable nociceptors versus non-irritable nociceptors whole exome sequence analysis

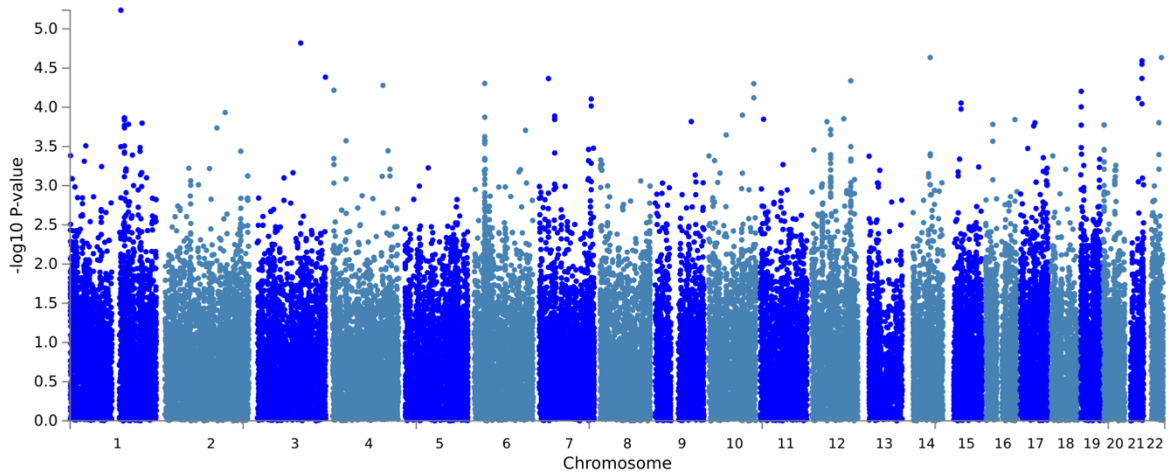

Gene-level associations of groups of rare variants for irritable nociceptors versus non-irritable nociceptors. We identified 1 variant with significance exceeding $P < 5 \times 10^{-5}$ (rs113126882 near the *NBPF13P [NBPF Member 13, Pseudogene]* gene, Odds Ratio = 0.27, $p = 5.8 \times 10^{-6}$).

Supplementary figure 11. Sensory loss vs hyperalgesia whole exome sequence analysis

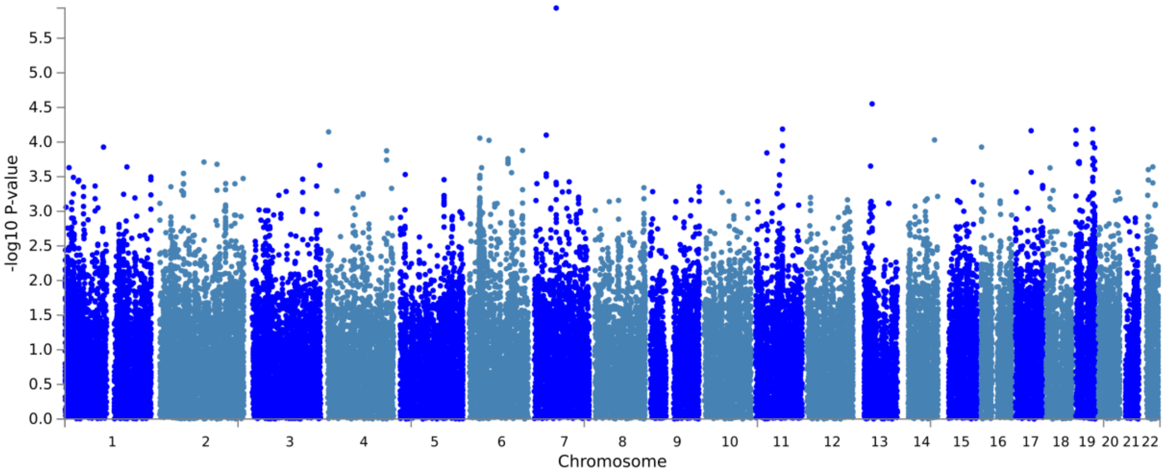

Gene-level associations of groups of rare variants for sensory loss vs hyperalgesia. We identified 1 variant with significance exceeding $P < 5 \times 10^{-5}$, with the top variant located near the *ZNF679 (Zinc Finger Protein 679)* gene (rs10949885, Odds Ratio = 0.57, $P = 1.31 \times 10^{-6}$).

Supplementary figure 12. KCNT2 variant rs114159097 *carrier QST profiles*

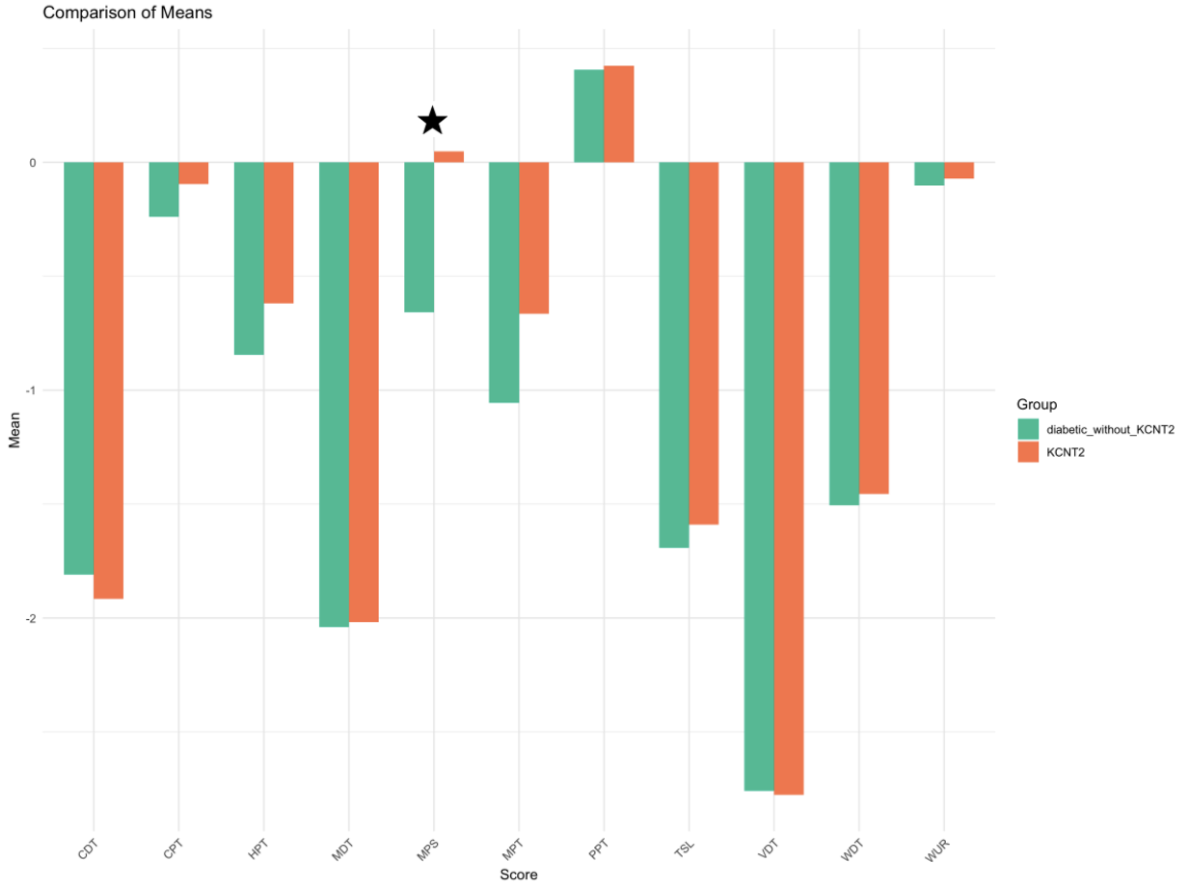

*Indicated significant result between KCNT2 (rs114159097 variant) carriers and diabetic polyneuropathy patients without this variant

CDT: Cold Detection Threshold
CPT: Cold Pain Threshold
HPT: Heat Pain Threshold
MDT: Mechanical Detection Threshold
MPS: Mechanical Pain Sensitivity
MPT: Mechanical Pain Threshold
PPT: Pressure Pain Threshold
TSL: Thermal Sensory Limen
VDT: Vibration Detection Threshold
WDT: Warm Detection Threshold
WUR: Wind-up Ratio

Supplementary Table 1. Detail of the cohorts included in the genetic analyses.

Study title/Cohort	Description of study	GWAS	WES	Participating sites	Study participant numbers		Online Database trial registration	Recruitment dates	Previous publications, including study description (PMID reference)
					Diabetes	Non-diabetes			
DOLORisk	Multi-centre, cross-sectional, observational study	✓	✓	Europe	1004	597	NCT04888455	June 2015 – June 2019	30756091
IDNC	Multi-centre, cross-sectional, observational study	✓	✓	Denmark	175	63	NCT02947828	October 2016 – December 2018	32909392, 31222961, 35176062
GeNeup	Multi-centre, cross-sectional, observational study	✓	✓	Norway	75	114	NCT03862365	August 2018 – December 2019	n/a
PROPANE	Multi-centre, cross-sectional, observational study	✓	✗	Italy and Netherlands	540	111	NCT02243475	September 2014 – September 2016	24776970, 25142720, 31041876
HIV-POGO	Single-centre, cross-sectional, observational study	✓	✗	London, UK	0	148	NCT02555930	December 2014 – January 2019	31855944, 36116766
OPTION - DM	Multi-centre randomised drug trial	✓	✗	UK	61	0	ISRCTN17545443	November 2017 – July 2019	36007534

IDNC: International Diabetes Neuropathy Consortium
GeNeup: Exploring the genetics of neuropathic pain study
PROPANE: Probing the Role of Sodium Channels in Painful Neuropathies
HIV-POGO: Clinical Phenotyping and Genotyping of HIV-Associated Sensory Neuropathy: The HIV-POGO Study (HIV-POGO)
GWAS: Genome Wide Association Study
WES: Whole exome sequencing
Total number of participants recruited before exclusions
IDNC and GeNeuP were included as part of DOLORisk extended cohort as clinical phenotyping was fully aligned.

Supplementary table 2. Description of the different aetiologies of neuropathic pain included in the study.

Aetiology	Diagnostic criteria
Diabetic polyneuropathy	Patient with a known diagnosis of diabetes mellitus and distal symmetrical polyneuropathy.[26]
Distal symmetrical polyneuropathy	Diffuse length dependant process - symptoms and/or signs in the feet that progress in proximal direction ("glove and stocking distribution") with abnormal nerve conduction studies to confirm diagnosis. Idiopathic and secondary causes, e.g. alcohol, vitamin B12 deficiency, included; but, diabetes and chemotherapy not included.
Small fibre neuropathy	Probable—symptoms in hands and feet consistent with small fibre dysfunction (pain and altered temperature sensibility), clinical signs of small fibre damage (reduced pin prick sensitivity and ability to discriminate warm/cool) and normal nerve conduction studies. Definite—symptoms in hands and feet, clinical signs of small fibre damage, normal nerve conduction studies, and altered intra-epidermal nerve fibre density at the ankle and/or abnormal quantitative sensory testing of thermal thresholds at the foot.[26]
Post-surgical nerve injury	Nerve injury after surgery (mastectomy and thoracotomy) with clinical evidence of nerve injury in the neuroanatomical distribution of neuropathic pain or other symptoms e.g., numbness.
Post traumatic neuropathy	Traumatic nerve injury with clinical evidence of nerve injury in the neuroanatomical distribution of neuropathic pain
Chemotherapy induced polyneuropathy	Peripheral neuropathy caused by chemotherapy.
Neuropathic pain NOS	Pain with a distinct neuroanatomically plausible distribution; however, no evidence of nerve injury found on clinical examination or specialised investigations
**Extreme phenotypes[27]**	
Erythromelalgia	Pain and erythema of the extremities which is exacerbated by warming and relieved by cooling. Initially episodic but may become persistent.
Familial episodic pain syndrome	Severe episodic pain usually localised to the trunk and limbs with no structural cause. Triggers include cold environment, exercise and fasting.
Insensitivity to pain	Inability to perceive painful stimuli. Other somatosensory modalities may be impaired but the predominant clinical presentation is loss of pain sensibility

Supplementary Table 3. Candidate genes for analysis in neuropathic pain

	Gene symbol	HGNC no.	Linked clinical condition	Altered pain in model organism?	Rare or common variant	OMIM	If known gain or LOF	Human Pain genetics DB	PUBMED Ids
1	*SCN9a*	10597	CIP, EM, PEPD, SFN, NP	Yes	Both rare and common	243000, 167400, 133020	LOF (CIP) GOF (EM, PEPD, SFN, NP)	Yes: MPD, Nocic, FM	14985375, 17145499
2	*SCN10a*	10582	SFN	Yes	Rare	615551	GOF	No	23115331, 26711856
3	*SCN11a*	10583	CIP, SFN	Yes	Rare	615548 615552	GOF (for both CIP and SFN)	No	23115331, 24036948
4	*KCNA1*	6218	NeuP	Yes	Rare and common	160120	LOF (dom negative)	No	25599232, 24578548
5	*KCNS1*	6300	NI, Sc	Yes	Common		Not known	Yes: NeuP	20724292
6	*P2RX7*	8537	PSP	Yes	Common		LOF		22447075, 24934217, 25472448
7	*NGF*	7808	CIP (HSAN5)	Yes	Rare	608654	LOF		14976160, 20978020
8	*NTRK1*	8031	CIP (HSAN4)	Yes	Rare	608654	LOF		8696348, 11668614
9	*SPTLC1*	11277	HSAN1	Yes	Rare	162400	GOF		11242106, 15037712
10	*SPTLC2*	11278	HSAN1	UK (not in PGD)	Rare	162400	GOF		12207934, 20920666
11	*WNK1*	14540	HSAN2	Yes	Rare	201300	LOF		15060842, 18521183

12	*GCH1*	4193	Sc	Yes	Common		LOF		25599448, 17057711
13	*TRPA1*	497	FEPS, NeuP	Yes	Rare and common	615040	GOF		20547126, 21468319
14	*TRPV1*	12716	NeuP	Yes	Common		UK		21468319
15	*PRDM12*	13997	CIP	UK (not in PGD)	Rare	616488	LOF		26975306, 26220135
16	*NAGLU*	7632	PN	UK (not in PGD)	Rare	616491	LOF		25818867
17	*COMT*	2228	Sc	Yes	common		LOF		22337560
18	*SETDB2*	20263	CIP*	Yes*	Rare		UK		Unpublished*
19	*OPRM1*	8156	PN	Yes	Common		UK		19303332
20	*Il6*	6018	Sc	Yes	Common		UK		15733644
21	*CACNG2*	1406	PSP	Yes	Common		UK		20688780
22	*MMP1*	7155	Sc	UK, (not in PGD)	Common		UK		23370084
23	*Chrna6*	15963	PSP	Yes	Common		UK		25972004
24	*TNF*	11892	PN	Yes	Common		UK		25756557
25	*SLC6A4*	11050	TN	Yes	Common		UK		24950698 12533631
26	*KCNA2*	6220	Nil	Yes	NA		UK		23792947, 23792947
27	*KCNA6*	6225	Nil	Yes	NA		NA		27033551
28	*KCNK18*	19439	Mig	Yes	Rare	613616	LOF		23994814, 20871611
29	*KCNK4*	6279	Nil	Yes	NA		NA		23994814
30	*HCN1*	4845	Nil	Yes	NA		NA		23549867
31	*HCN2*	4846	Nil	Yes	NA		NA		21903816
32	*HCN3*	19183	Nil	Yes	NA		NA		18958363
33	*HCN4*	16882	Nil	Yes	NA		NA		18958363
34	*SCN1A*	10585	Nil	Yes	NA		NA	Yes: OF	27281198

35	*SCN3A*	10590	Nil	Yes	NA		NA		15152043
36	*SCN8A*	10596	TN	Yes	Rare		GOF		22075254 27496104
37	*ADRB2*	286	CWP	Yes	Common		UK		20167428
38	*IL10*	5962	PSP	Yes	Common		UK		24411993
39	*Il1R2*	5994	PSP	UK (not in PGD)	Common		UK		2441193
40	*KCND2*	6238	PSP	Yes	Common		UK		16600858, 25599232
41	*Piezo2*	26270	DAIPT	Yes	Rare	617146	GOF		27653382, 23575686
42	*SCN2A*	10588	EIEE	UK (not in PGD)	Rare	613721	LOF		20956790
43	*TRPM8*	17961	Mig	Yes	Common		NA		23407943, 27322543
44	*PRKCA*	9393	NeuP (PSP)	Yes	Common		UK		22042410, 28051079
45	*ZSCAN20*	13093	PN	No	Common		UK	No	26629533

Clinical conditions: CIP=congenital insensitivity to pain, CWP=chronic widespread pain, DAIPT=arthrogryposis with impaired proprioception and touch, EIEE= early infantile epileptic encephalopathy, EM= erythromelalgia, FM= fibromyalgia, HSAN= hereditary sensory and autonomic neuropathy, Mig=migraine, MPD= Mendelian pain disorder, NeuP= neuropathic pain, NI=nerve injury, Noci=nociception, OF=orofacial, PEPD= paroxysmal extreme pain disorder, PN=painful neuropathy, PSP=post-surgical pain, Sc=sciatica, SFN= small fibre neuropathy, TN=trigeminal neuralgia

UK= Unknown

*unpublished

Supplementary table 6. PheWAS associations of KCNT2 with Diverse Traits

Genes	PMID	Domain	Trait	P-value
KCNT2	26691988	Ophthalmological	Age-related Macular Degeneration	3.50E-65
	23455636	Ophthalmological	Age-related Macular Degeneration	3.54E-42
	23455636	Ophthalmological	Neovascular disease	1.52E-35
	23455636	Ophthalmological	Geographic atrophy	1.41E-20
	28240269	Cell	HPX - Hemopexin	6.42E-20
	20385819	Ophthalmological	Age-related Macular Degeneration	1.21E-15
	28240269	Cell	CFH - Complement factor H	4.98E-11
	31427789	Psychiatric	Morning/evening person (chronotype)	1.23E-10
	30696823	Psychiatric	Chronotype	3.71E-10
	30804565	Psychiatric	Morningness	1.42E-09
	26955885	Psychiatric	Chronotype (continuous)	1.08E-08
	26955885	Psychiatric	Extreme chronotype	2.23E-08
	27494321	Psychiatric	Chronotype	3.89E-08
	30696823	Psychiatric	Morning person (binary)	1.03E-07
	31427789	Metabolic	Comparative body size at age 10	7.55E-07
	31427789	Psychiatric	Daytime dozing / sleeping (narcolepsy)	3.50E-06
	27864402	Activities	Self-rated health	4.86E-06

Supplementary table 7. PheWAS associations of NOS1AP with Diverse Traits

Genes	PMID	Domain	Trait	P-value
NOS1AP	31217584	Immunological	White blood cells	4.41E-86
	31217584	Cardiovascular	QT interval	2.12E-22
	30598549	Skeletal	Estimated bone mineral density from heel ultrasounds	8.99E-11
	30048462	Skeletal	Heel bone mineral density	3.13E-10
	BioRxiv: https://doi.org/10.1101/288623	Neurological	Average across all tracts mode of anisotropy	1.28E-09
	31427789	Skeletal	Standing height	1.38E-09
	30804566	Neurological	Frequent insomnia symptoms	2.16E-07
	31427789	Psychiatric	Smoking status: Never	7.89E-07
	28240269	Cell	ETHE1 - Persulfide dioxygenase ETHE1, mitochondrial	1.71E-06
	27863252	Immunological	Immature fraction of reticulocytes (two-way meta)	4.67E-06
	31427789	Skeletal	Sitting height	6.18E-06
	27863252	Immunological	Monocyte percentage of white cells (three-way meta)	6.61E-06
	30239722	Metabolic	Body Mass Index	6.67E-06
	31427789	Psychiatric	Anxiety - Ever felt worried, tense, or anxious for most of a month or longer	7.36E-06
	24934506	Endocrine	Skin fluorescence	7.55E-06
	28240269	Cell	MSLN - Mesothelin	8.91E-06
	30124842	Skeletal	Height	9.28E-06

Supplementary Table 10. Rare variant association analysis

EMMAX test accounting for related individuals								
Gene ID	Total variants	Pass variants	Burden Count	P.value	BETA	R2	Model	Test
SCN9A	12	12	73	0.0037	0.3343	0.005769	Whole cohort - model 1	Burden
Unrelated individuals only								
			Fraction of individuals with rare variants					
SCN9A	12	6	0.043	0.0033			Diabetics – model 1	Reverse regression
OPRM1	7	4	0.028	0.0037			Diabetics – model 2	Reverse regression

Supplementary Table 11. Variants driving the significant gene-wise association for the SCN9A gene. In the ClinVar column when conflicting interpretations we show the number of submissions supporting each class of pathogenicity in parentheses.

**Variant** *REFSeq annotation*	**Variant** *ENSEMBL annotation*	**AF GNOMAD NFE**	**AF in cohort**	**AF affected**	**AF un-affected**	**Clin Var**	**Nr of affected carriers**	**Pubmed ID**	**Electrophysiology and morphology characterisation**
p.Leu1267 Val	p.Leu1278 Val* c.3832C>G	0.0021	0.002036	0.00293	0.00114	Conflicting classifications of pathogenicity Uncertain significance(1) ; Likely benign(11)	6	*PMID*: 25250524, 24776970.	**N/A**. (the mutation does not seem to confer hyperexcitability in DRGs when assessed by electrophysiology, unpublished data, Huang et al., Brain, 2014).
p.Asn1245 Ser	p.Asn1256 Ser* c.3767A>G	0.0075	0.007563	0.00927	0.00572	Conflicting classifications of pathogenicity Uncertain significance(5) ; Benign(6); Likely benign(9)	19	*PMID*: 29264398, 28440294, 28235406, 25250524, 23895530.	**N/A**.
Val991Leu	p.Val1002L eu c.3004G>T	0.0027	0.004072	0.00439	0.00114	Pathogenic.	9	*PMID*: 23450472, 21698661, 23280954,	**GOF**. Enhanced resurgent currents and DRGs hyperexcitability (depolarised RMP, reduced current threshold, increased SA although not significant (when

									assessed with M932L variant, Faber et al. Ann. Neurol, 2011)).
p.Ile739Val	p.Ile750Val c.2248A>G	0.0035	0.00349	0.00390	0.00343	Conflicting classifications of pathogenicity Uncertain significance(1); Benign(2); Likely benign(12)	8	*PMID*: 25250524, 22826602.	**GOF**. Impaired channel slow inactivation and depolarised RMP in DRGs (Han et al, Brain, 2012).
p.Ile229Met	p.Ile228Met c.684C>G	0.0017	0.001745	0.00293	0.00000	Conflicting classifications of pathogenicity Uncertain significance(15); Benign(2)	6	*PMID*: 25250524, 22136189, 30316835, 25993546, 23280954, 30554136, 27525141, 21698661.	**GOF**. Impaired slow inactivation with depolarised $V_{1/2}$ in HEK293 cells (Faber et al. Ann. Neurol, 2011; Estacion et al, Mol Pain, 2011); depolarised RMP, unchanged current threshold, increased firing frequency and SA in DRGs (Estacion et al, Mol Pain, 2011). Decreased nerve density in Zebrafish embryos, increased activity at elevated temperature in

									Zebrafish larvae (Eijkenboom at et al., Experimental Neurology, 2018). 20% reduction in neurite length in DRGs (Persson et al, Annals of Neurology, 2012). CM I228M fibres have slower conduction velocities than controls, conduction velocity of CMi I228M nociceptors are lower than controls, although not statistically different (Namer et al, Pain, 2015).
p.Arg185His	p.Arg185His c.554G>A	0.0025	0.002327	0.00293	0.00114	Benign/Likely benign	6	*PMID*: 30569495, 29176367, 27843123, 25316021, 23895530, 25250524, 24817410, 22035805, 22826602, 21698661,	**GOF**. Enhanced resurgent currents in DRGs (Han et al, Brain, 2012; Faber et al. Ann. Neurol, 2011), unaltered RMP (Han et al, Brain, 2012).

								30554136, 27525141.	
p.Tyr990Cys	p.Tyr1001Cys* c.3002A>G	0.00086	0.0005817	0.00049	0.00000	Conflicting classifications of pathogenicity Uncertain significance(5); Benign(2); Likely benign(5)	1	*PMID*: 30554136.	**N/A**.
p.Ile720Lys	p.Ile731Lys c.2192T>A	0.00019	0.0005817	0.00098	0.00000	Conflicting classifications of pathogenicity Uncertain significance(8); Likely benign(6)	2	*PMID*: 21698661, 22035805, 25250524, 27525141, 23280954.	**GOF**. Impaired slow inactivation in HEK293 cells with a depolarized V1/2; depolarizing shift in RMP and increased excitability (43% reduction in current threshold) in DRGs, alongside increased number of APs and trend towards increase SA (although not statistically significant, Faber et al. Ann. Neurol, 2011). 720K variants displayed a trend toward reduced

									neurite length in DRGs (6%, although not statistically significant, Persson et al, Annals of Neurology, 2012).
p.Arg372His	p.Arg372His* c.1115G>A	0.000046	0.0005817	0.00098	0.00000	Uncertain significance	2	*N/A.*	**N/A.**
p.Tyr367His	p.Tyr367His* c.1099T>C	0.00025	0.0005817	0.00049	0.0011	Uncertain significance	1	*N/A.*	**N/A.**
p.Lys1387Gln	p.Lys1387Gln*	0.000017		0.00049	0.00000	N/A	1	*N/A.*	**N/A.**
p.Ser461Leu	p.Ser459Leu* c.1379C>T	0.00029		0.00049	0.00000	Uncertain Significance.	1	*N/A.*	**N/A.**

* Novel *SCN9A* variants.

RMP: resting membrane potential.
SA: spontaneous activity.
CM: mechanoresponsive.
CMi: mechanoinsensitive

Supplementary Table 12. Variants driving the significant gene-wise association for the OPRM1 gene. In the ClinVar column when conflicting interpretations we show the number of submissions supporting each class of pathogenicity in parentheses.

Variant REFSeq annotation	Variant ENSEMBL annotation	AF GNOMAD NFE	AF in cohort	AF affected	AF un-affected	Clin Var	Nr of affected carriers	Pubmed ID	Electrophysiology and morphology characterisation
p.Ser147Cys	p.Ser147Cys c.440C>G	0.0074	0.0058	0.00682	0.00457	Benign.	13	*PMID*: 19528663, 16476706, 9399694, 28333316, 23454283.	**N/A.** Increased potency for morphine (small leftward shift in EC_{50}) in HEK293 cells (Ravindranathana et al., PNAS 2009).
p.Arg181Cys	p.Arg181Cys c.541C>T	0.0023	0.0012	0.00146	0	N/A	3	*PMID*: 27113810, 30969062, 19528663, 28333316.	**N/A.** Significantly reduced response to opioids (Skorpen et al, Acta Anaest. Scand. 2016;).
p.Cys192Phe	p.Cys192Phe c.575G>T	0.0072	0.0073	0.00780	0.00457	Likely benign	16	*PMID*: 19528663, 28333316, 23454283, 29888403.	**N/A.** Decreased potency for morphine and DAMGO (rightward shift in EC_{50}) in HEK293 cells (Ravindranathana et al., PNAS 2009).
p.Arg260His	p.Arg260His c. 779G>A	0.00062	0.0015	0.00243	0	N/A	4	*PMID*: 11457836, 22547174.	**N/A.** Basal MOR signalling is

									significantly reduced (Wang et al., JBC 2001).
